# Characterizing the diversity and distribution of tropical coastal blue carbon using environmental DNA

**DOI:** 10.1016/j.isci.2025.113837

**Published:** 2025-10-24

**Authors:** Wei Jie Dennis Tan, Jia Jin Marc Chang, Valerie Kwan, Danwei Huang

**Affiliations:** 1 Department of Biological Sciences, National University of Singapore, 16 Science Drive 4, Singapore 117558, Singapore; 2 Lee Kong Chian Natural History Museum, National University of Singapore, 2 Conservatory Drive, Singapore 117377, Singapore; 3 Centre for Nature-based Climate Solutions, National University of Singapore, 16 Science Drive 4, Singapore 117558, Singapore; 4 Tropical Marine Science Institute, National University of Singapore, 18 Kent Ridge Road, Singapore 119227, Singapore

**Keywords:** Earth sciences, Biogeochemistry, Aquatic science, Global carbon cycle, Aquatic biology, Biogeoscience

## Abstract

Tropical coastal blue carbon is stored in shallow-water ecosystems such as mangrove forests, seagrass meadows, and macroalgal beds. Carbon is also exported from these ecosystems offshore and into sediment pools as cellular organic carbon that comprises DNA, among other components. Environmental DNA (eDNA) has recently shown promise in the identification and quantification of macrophytes from coastal sediment and seawater. Here, we test an eDNA approach for detecting and characterizing tropical blue carbon, uncovering significant seasonal patterns in tropical coastal macrophyte communities with high site fidelity. Results underscore important environmental constraints on eDNA monitoring of tropical blue carbon, including high temperatures and rainfall, which can impact eDNA residence time and detection levels. There remains a critical need to improve reference DNA databases and protocols to more precisely characterize macrophyte communities. These findings contribute to the development of an eDNA-based monitoring system to track nearshore stocks and fluxes of tropical blue carbon.

## Highlights

• eDNA is promoted as a promising tool for identifying and quantifying macrophyte blue carbon
• 18S rRNA metabarcoding identified 27 macrophyte taxa from seawater and sediment
• Macrophyte eDNA displayed high site fidelity but limited depth difference
• Mangrove, seagrass, and green algal eDNA are limited by environment and primer biases

## Graphical abstract

**Figure:**
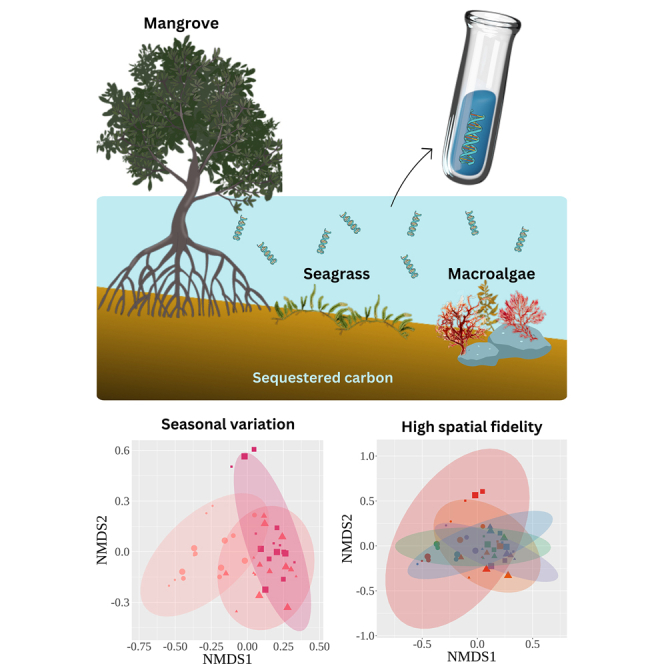

## Introduction

Natural climate solutions have risen in prominence as instruments for mitigating climate change.^1^^,^^2^ Blue carbon, defined as coastal habitats that can sequester and store significant amounts of organic carbon in the long term, has shown great potential as a form of nature-based climate solution.^3^ These habitats include angiosperm-dominated coastal ecosystems—mangrove forests, seagrass meadows, and tidal marshes—with organic carbon stored within both live plant tissue and sediment around the rooted vegetation. The sediment has a considerably larger capacity for storing more permanently sequestered carbon,^4^^,^^5^ and comprises both autochthonous carbon produced within an ecosystem and allochthonous carbon transported from external sources into an ecosystem.^6^ Allochthonous carbon can amount to a substantial proportion of sediment carbon and may be derived from a variety of carbon donors such as other coastal angiosperms and macroalgae.^6^

The inclusion of macroalgae as blue carbon ecosystems, however, is currently debated.^7^^,^^8^ In contrast to traditional blue carbon ecosystems, macroalgae mostly grow on hard substrates and thus have limited ability to bury carbon locally, raising questions about their ability to act as long-term sinks.^7^^,^^9^ However, the climate mitigation potential of macroalgae is immense. First-order estimates of macroalgal carbon sequestration place it at 173 TgC y^−1^ globally, potentially more than mangroves, tidal marshes, and seagrasses combined due to its high productivity and extent.^9^^,^^10^ Even then, the potential of macroalgae lies in their role as a carbon donor. An estimated 43% of macroalgal net primary productivity—the net amount of CO_2_ absorbed from photosynthesis after deducting CO_2_ released from respiration—is exported, contributing a colossal 679 TgC yr^−1^ of global carbon flux.^9^ Of this, 24.4% is either buried in shelf sediment or the deep sea.^9^

Macroalgal carbon has been detected in both surface and subsurface marine sediment across depths and biogeographic regions, and has been traced using stable carbon isotopes and lipids, sterols, and carotenoids.^9^ Benthic macroalgae have also been reported to be the source of oil deposits, indicative of their ability to sequester carbon over centennial timescales.^9^^,^^11^^,^^12^^,^^13^ Evidence from studies using stable isotopes to trace macroalgal contributions to sediment has found that 2–9% of carbon from *Gracilaria* spp. is incorporated into lagoon sediment, and that macroalgal organic carbon contributes up to 14% of surface sediment organic carbon in seagrass meadows, even more than seagrasses themselves.^14^^,^^15^ Given the significant potential of macroalgae in sequestration and the role they play as carbon donors, the inclusion of macroalgae in blue carbon research is warranted.

While sound management of blue carbon ecosystems is key to maximizing their climate mitigation capacity, our lack of understanding of interconnectivity and carbon flows between these ecosystems limits their applications for carbon crediting and management.^16^ Blue carbon ecosystems are situated at the interface between land and sea, with high rates of transfer of organic matter and genetic material from both terrestrial and oceanic sources up to hundreds of kilometres away in the form of detritus or dissolved carbon.^16^^,^^17^ Allochthonous carbon from carbon donors (also known as carbon sources) is important to track, but current methods of tracing carbon sources are inadequate and cannot distinguish between different blue carbon sources.^10^^,^^18^ Knowledge of such carbon sources and fluxes among various blue carbon ecosystems is critical to provide policymakers and managers with the ability to judiciously allocate limited resources to enhance and maintain key local blue carbon ecosystems.^18^ An improved understanding of carbon flows within marine ecosystems provides an opportunity for the identification and conservation of habitats that contribute to blue carbon ecosystems and more accurate carbon accounting.^10^^,^^16^^,^^18^

Established approaches to tracing carbon provenance involve assessing the bulk properties of soils. The testing of carbon and nitrogen elemental and stable isotopic composition has been widely conducted to study sources of organic matter in blue carbon ecosystems.^18^ This method has so far proven useful for sources of blue carbon with distinct isotopic ratios such as seagrasses, mangroves, and macroalgae.^19^^,^^20^ For example, mixing models based on measurements of δ^13^C, δ^15^N, and bulk organic C:N ratio can estimate the relative contributions of oceanic, terrestrial, mangrove, and seagrass carbon sources to suspended particulate matter and sediment trap samples from mangrove bays of Phuket, Thailand.^21^ Such models were also able to identify the relative contributions of various functional biota—plants, microphytobenthos, phytoplankton, and macroalgae—to sediment organic matter in the *Zostera noltii* meadow of Arcachon Bay, France.^22^ However, elemental and stable isotopic analyses require precise knowledge of potential source values—which may change given site-specific soil composition—and sources to have significantly different values to be distinguishable.^18^^,^^20^ Meeting either requirement is complex, as isotopic values for blue carbon ecosystems may overlap or vary given different species, organic materials, microhabitats, seasons, and growth cycles.^18^^,^^23^ Indeed, marine suspended particulate organic matter, different species of seagrasses, and terrestrial plants display δ^13^C signatures that are indistinguishable using conventional mixing models, whereas metabarcoding of environmental DNA (eDNA) can be used to profile the presence of specific seagrass and terrestrial plant species in sediment cores.^24^

Environmental DNA (eDNA), which comprises genetic material in the form of excreted cells and tissues, microorganisms, or leaked extracellular material, displays great promise in the ecosystem-wide detection and monitoring of blue carbon and complements traditional techniques such as field surveys and quantifying bulk properties of soils.^18^^,^^25^^,^^26^ High-throughput sequencing and metabarcoding of eDNA have already been widely used in recent years to assess the presence and abundance of marine fauna and cryptofauna,^27^^,^^28^^,^^29^^,^^30^ but applications on marine macrophytes remain limited, forming less than 1.5% of eDNA and eRNA studies in aquatic community ecology.^8^^,^^18^^,^^31^^,^^32^^,^^33^^,^^34^^,^^35^ Given that approximately 3% of cellular organic carbon is DNA, blue carbon eDNA could provide a rough estimate of autochthonous and potentially allochthonous carbon stocks from adjacent ecosystems.^36^

Positive relationships between species biomass and abundance and eDNA concentration in water have been found in multiple studies.^37^^,^^38^^,^^39^^,^^40^^,^^41^^,^^42^^,^^43^^,^^44^^,^^45^^,^^46^ eDNA also degrades rapidly in marine environments—within hours up to two days—which increases the probability that eDNA collected at inshore environments originates from local blue carbon ecosystems.^30^^,^^47^^,^^48^ This is especially so in tropical ecosystems, given the higher degradation rate of eDNA in warmer waters, resulting in a decrease in eDNA detection by 1.67 times for every 1.02°C increase in temperature.^49^^,^^50^ Provided that DNA sequences for targeted blue carbon species are available, and depending on the resolution of metabarcoding markers used, eDNA can also be used to identify blue carbon sources at specific taxonomic levels. Such data provide opportunities for taxon-specific calculations of carbon stock when complemented with other methods for assessing abundance in blue carbon ecosystems.^18^^,^^30^

The combination of high potential specificity and high site fidelity makes eDNA an attractive candidate for use in developing real-time monitoring systems of blue carbon ecosystem diversity and conservative approximations of carbon stock.^25^^,^^51^ eDNA is also able to track organismal movement across habitats, and could potentially contribute to our understanding of carbon flows on a local scale by matching eDNA to carbon sources in situations whereby inventories of nearby blue carbon ecosystems are available.^18^^,^^52^^,^^53^

**Figure 1:**
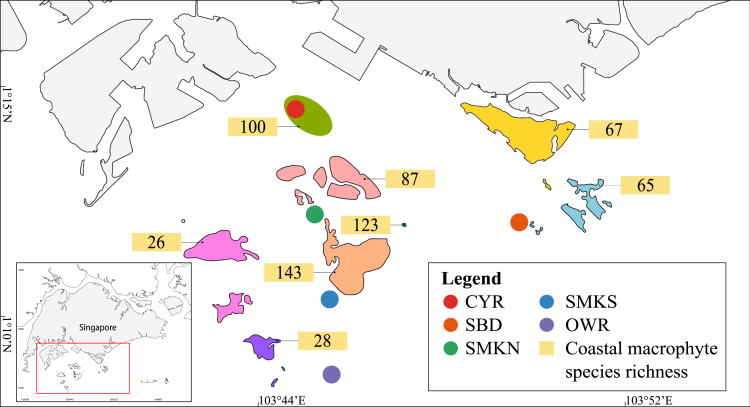
Map of sampling sites and surrounding areas, with published estimates of coastal macrophyte species richness Sampling sites: CYR (Cyrene Reefs), SBD (Pulau Subar Darat), SMKN (Pulau Semakau North), SMKS (Pulau Semakau South), and OWR (Open Water).

This study tested an eDNA approach for characterizing tropical coastal macrophyte richness, diversity, and relative abundance. eDNA samples were obtained from Singapore (1°09′*N*–1°29′N, 103°36′E−104°06′E) (Figure 1), which has an equatorial climate characterized by high annual temperature and rainfall, with two annual monsoon seasons—the dryer southwest (SW) monsoon (June–September), and the wetter northeast (NE) monsoon (December–March)—separated by two inter-monsoon periods. Mangroves, seagrasses, and macroalgae are found in Singapore, largely along the northern coastline and offshore islands in the northeast and southwest of the mainland.^54^^,^^55^ The spatial extents of these coastal macrophytes constitute 8.1 km^2^ of mangrove forests, 2.2 km^2^ of seagrass meadows, and 5.9 km^2^ of macroalgal beds.^54^^,^^56^^,^^57^^,^^58^ Given Singapore’s location within a marine biodiversity hotspot, relatively high species diversity has been recorded, including 35 mangrove species (around half of global diversity) and 13 seagrass species (around a fifth of global diversity).^59^^,^^60^^,^^61^ For macroalgae, 286 species representing around 2.5% of global diversity have been recorded in Singapore, corresponding to the generally lower macroalgal diversity in the tropics.^54^^,^^62^

Seasonal, spatial, and depth-wise variations in eDNA levels of tropical coastal macrophytes were examined by sampling eDNA in water at 1 m, 10 m, and 20 m as well as sediment at five sites over an area of 41.9 km^2^ (Figure 1). The 18S-v7 mini-barcode was used to identify and estimate the composition of eDNA from seagrasses, mangroves, and macroalgae in coastal seawater and sediment taken from sites close to blue carbon ecosystems. While past studies on eDNA detection of blue carbon in temperate regions exist, findings from this study add to the hitherto limited body of work on the use of eDNA to detect blue carbon in the tropics and represent a baseline in equatorial waters where a unique mixture of environmental conditions complicate eDNA detection.

## Results

### Sample collection

A total of 45 water samples and 15 sediment samples (excluding negative controls) were collected from five sites—Cyrene Reefs (CYRs), Pulau Subar Darat (SBD), Pulau Semakau North (SMKN), Pulau Semakau South (SMKS), and Open Water (OWR) (Figure 1; Table S1). Total organic carbon (TOC) analysis generally detected higher organic carbon concentrations during the northeast monsoon as compared to the southwest monsoon (Figures S1 and S2). Little or inconsistent variation was found between sites and depths. There were no significant linear relationships between TOC and abundance of macrophyte eDNA reads in water samples (Figure S3), as well as between water TOC and sediment TOC (Figure S4).

Successful amplification was achieved for all five replicates of each water sample. However, only sediment samples from the first sampling season (33.3% of sediment samples) were successfully amplified; PCR of the remaining sediment samples was unsuccessful despite repeated attempts. In total, 250 sample amplicons were successfully sequenced.

### Environmental DNA detection of blue carbon taxa

A total of 1,092,488,209 pairs of raw Illumina sequencing reads were obtained, of which 937,118,334 (88.1%) were successfully merged and 780,201,326 paired reads (84.0%) were successfully demultiplexed into replicates. After filtering, 6,701 unique sequences were generated from 17,809,887 reads (2.3%). Of these, 171 MOTUs were delimited at a 4% threshold. Of the remaining 144 unassigned MOTUs, 129 did not have matches to the NCBI *nt* database above the BLAST threshold of 80%, and 15 were not matched to a macrophyte lineage or were non-marine. For instance, there were MOTUs matched to green and red microalgae (but not macroalgae) that were removed from the list of identified MOTUs. Overall, 15.8% of the 171 MOTUs were assigned a taxonomic rank of a macrophyte lineage at ≥80% identity, resulting in a final list of 27 MOTUs from 164,988 reads (Table 1). Two-thirds of identified MOTUs (18) could be identified to genus based on matches, while the other nine MOTUs would match only at ≥80% identity to family, class, or order.

**Table 1:** List of 27 identified MOTUs, their macrophyte lineage, assigned taxonomic level, scientific name, and match to recorded macrophyte species in Singapore

Lineage	Taxonomic level	Scientific name	Recorded in Singapore
Rhodophyta	Class	Florideophyceae sp. 1	–
Rhodophyta	Class	Florideophyceae sp. 2	–
Rhodophyta	Order	Corallinales sp.	–
Rhodophyta	Order	Gigartinales sp.	–
Rhodophyta	Family	Erythrotrichiaceae sp.	No
Rhodophyta	Family	Peyssonneliaceae sp.	Yes
Rhodophyta	Family	Rhodomelaceae sp.	Yes
Rhodophyta	Genus	*Asparagopsis* sp.	Yes
Rhodophyta	Genus	*Centroceras* sp.	Yes
Rhodophyta	Genus	*Champia* sp.	Yes
Rhodophyta	Genus	*Chondria* sp.	Yes
Rhodophyta	Genus	*Colaconema* sp.	Yes
Rhodophyta	Genus	*Crustaphytum* sp.	No
Rhodophyta	Genus	*Gloiocladia* sp.	No
Rhodophyta	Genus	*Hypnea* sp.	Yes
Rhodophyta	Genus	*Lomentaria* sp.	No
Rhodophyta	Genus	*Lophocladia* sp.	Yes
Rhodophyta	Genus	*Portieria* sp. 1	Yes
Rhodophyta	Genus	*Portieria* sp. 2	Yes
Rhodophyta	Genus	*Predaea* sp.	No
Rhodophyta	Genus	*Pterocladiella* sp.	Yes
Rhodophyta	Genus	Stylonema sp.	No
Rhodophyta	Genus	*Titanoderma* sp.	No
Rhodophyta	Genus	*Wrangelia* sp.	No
Phaeophyceae	Class	Phaeophyceae sp.	–
Phaeophyceae	Family	Sargassaceae sp.	Yes
Phaeophyceae	Genus	*Dictyota* sp.	Yes

**Figure 2:**
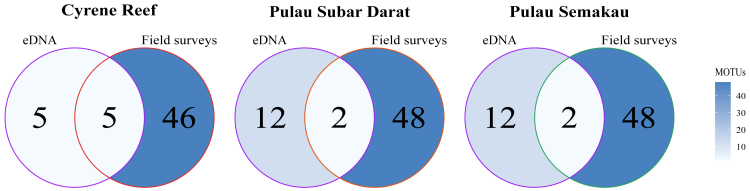
Comparison of identified MOTUs from eDNA and genera recorded from past field surveys at each study locality

Only red and brown macroalgae (Rhodophyta and Phaeophyceae, respectively) were unambiguously represented in the identified MOTUs across all sites (Table 1). No reads could be confidently assigned to mangrove or seagrass taxa. Rhodophyta was the more diverse of the two macroalgal groups, with 24 taxa (88.9%) identified across 20 families, 17 of which were at the genus level. Three taxa were identified from Phaeophyceae, with one identified to genus. Of the 27 identified taxa, 17 (63.0%) were matched at the genus or family level to taxa previously identified in Singapore. Matching taxa identified at the order level and above were deemed too uncertain. The macrophyte taxa detected were consistent between the NCBI *nt* and SILVA SSU Ref NR 99 databases. Eight taxa detected here have not previously been recorded in Singapore. However, a comparison of identified MOTUs at CYR, SBD, as well as SMKN and SMKS with macroalgal genera recorded from field surveys at Cyrene Reefs, Pulau Subar Darat, and Pulau Semakau, respectively, revealed weak overlaps between eDNA-identified taxa, with eDNA detecting only 20–28% of recorded genera (Figure 2).

### Taxon richness in water samples across season, site, and depth

**Figure 3:**
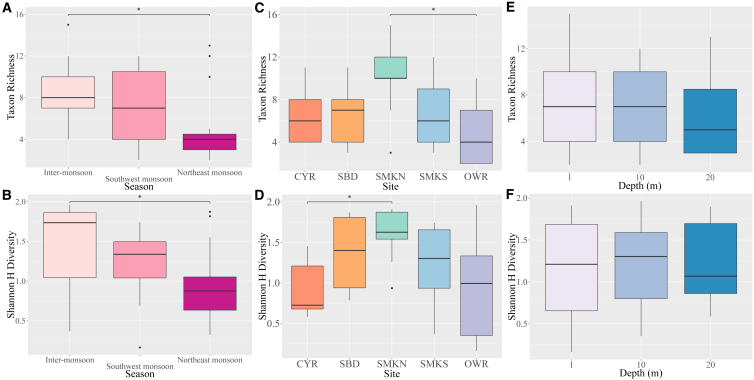
Variation in coastal macrophyte richness and Shannon diversity (A and B) Among seasons across all sites and depths. (C and D) Among sites across all seasons and depths. (E and F) Among depths across all sites and seasons. Data represented by median, first and third quartiles. Asterisks denote significant differences (*p* < 0.05, post-hoc Dunn's test).

Overall, taxon richness differed between seasons (Figure 3). Samples taken during the inter-monsoon season had the highest median richness at eight taxa, followed closely by the southwest monsoon at seven taxa. The northeast monsoon displayed the lowest median richness at four taxa. Kruskal-Wallis test indicated that this difference was significant (*χ*^2^ = 8.1201, *df* = 2, *p* = 0.01725). Post-hoc Dunn’s test detected a significant difference between the inter-monsoon and northeast monsoon (Z = 2.8263, *p*_adj_ = 0.01413), which was congruent with observed patterns (Figure 3). However, this trend was dissimilar among sites, with CYR and SBD peaking during the southwest monsoon, and SMKN having high richness during the northeast monsoon (Figure S5).

Taxon richness also varied between sites, with SMKN having the highest median taxon richness at 10 taxa, followed by SBD with seven taxa, both CYR and SMKS with six taxa, and OWR with four taxa (Figure 3). Kruskal-Wallis test indicated that this variation was significant (*χ*^2^ = 10.417, *df* = 4, *p* = 0.03396). Post-hoc Dunn’s test indicated that this result was driven by a significant difference between SMKN and OWR (Z = 3.1430, *p*_adj_ = 0.01672).

**Figure 4:**
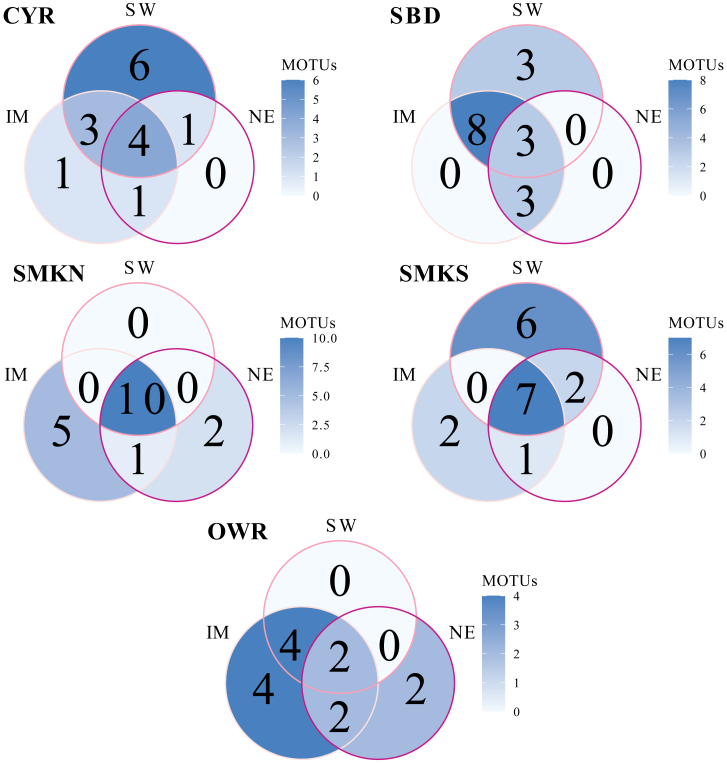
Comparison of MOTU composition among seasons at each study site SW = southwest monsoon, NE = northeast monsoon, and IM = inter-monsoon.

Comparisons of MOTU composition between sites found varying overlaps between sites across seasons, with a stronger taxon overlap between the inter-monsoon and southwest monsoon than with the northeast monsoon (Figure 4). Sites closer to each other were generally more similar in terms of MOTU composition, such as SMKN and SMKS.

There was a limited trend of taxon richness with depth, even as the deepest samples recovered the fewest taxa, with richness decreasing from seven taxa at 1 m and 10 m to five taxa at 20 m (Figure 3). However, the Kruskal-Wallis test indicated that this variation was not significant (*χ*^2^ = 0.51114, *df* = 2, *p* = 0.7745).

### Taxon diversity in water samples across season, site, and depth

Overall, Shannon diversity differed between seasons (Figure 3). Samples taken during the inter-monsoon had the highest median diversity score of 1.74, followed by the southwest monsoon and northeast monsoon at 1.34 and 0.876 respectively. Different sites also responded distinctly to seasonal changes that corresponded with changes in richness, with CYR and SMKS peaking in diversity during the southwest monsoon, and diversity at SMKN was higher during the northeast monsoon (Figure S2). The Kruskal-Wallis test indicated that seasonal differences significantly drove Shannon diversity (*χ*^2^ = 7.4443, *df* = 2, *p* = 0.02418). Post-hoc Dunn’s test detected a significant difference in Shannon diversity between the inter-monsoon and northeast monsoon (Z = 2.7246, *p*_adj_ = 0.01413) (Figure 3).

Shannon diversity also varied between sites, with SMKN having the highest median diversity of 1.63, followed by SBD, SMKS and OWR (Figure 3). Samples from CYR had the lowest diversity at 0.726, although OWR showed the greatest variation in diversity with the lowest lower quartile. The Kruskal-Wallis test indicated that this variation was significant (*χ*^2^ = 11.356, *df* = 4, *p* = 0.02284). Post-hoc Dunn’s test indicated that this difference was driven by the difference between SMKN and CYR (Z = −3.0329, *p*_adj_ = 0.01931).

For depth, median taxon diversity peaked at 10 m, with 20 m showing the lowest Shannon diversity (Figure 3). As with taxon richness, the Kruskal-Wallis test indicated that this variation was not significant (*χ*^2^ = 0.079614, *df* = 2, *p* = 0.961).

### Environmental DNA abundance in water samples across season, site, and depth

**Figure 5:**
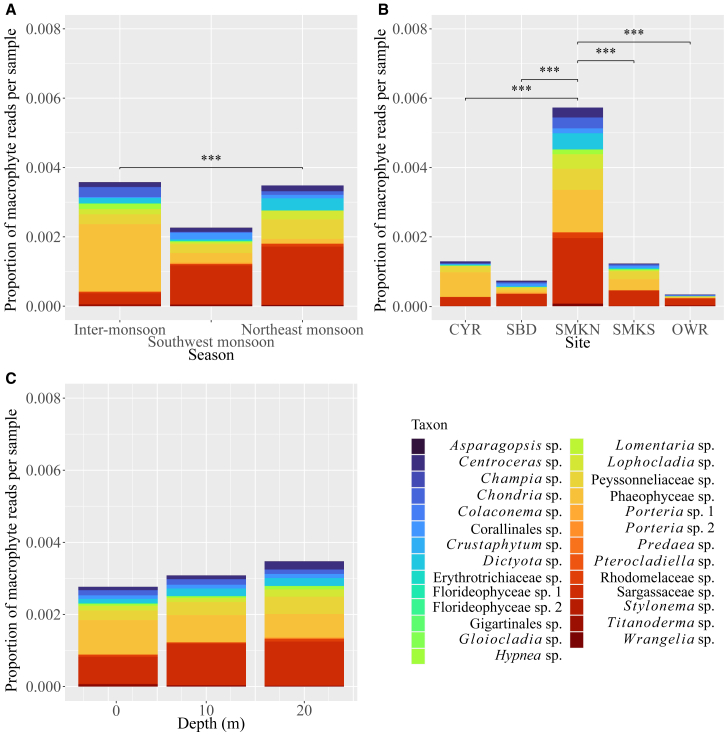
Cumulative relative abundance of macrophyte reads per sample (A) By season. (B) By site. (C) By depth. Asterisks denote significant differences (*p* < 0.001, post-hoc Dunn's test).

eDNA collected during the southwest monsoon showed the lowest macrophyte relative abundance, with a much smaller difference between inter-monsoon and northeast monsoon (Figure 5). Kruskal-Wallis test indicated differences in eDNA abundance between seasons were significant (*χ*^2^ = 14.081, *df* = 2, *p* = 0.0008758). Post-hoc Dunn’s test detected a significant difference in relative abundance between the northeast monsoon and the inter-monsoon (Z = 3.734, *p*_adj_ = 0.0005654), but not between the inter-monsoon and southwest monsoon (Z = 1.545, *p*_adj_ = 0.1222) or between both monsoons (Z = −2.088, *p*_adj_ = 0.1104).

SMKN had by far the highest macrophyte relative abundance (Figure 5), followed by CYR, SMKS, SBD, and lastly OWR. Kruskal-Wallis test indicated differences in relative abundance between sites were significant (*χ*^2^ = 34.314, *df* = 4, *p* = 0.0000006425). Post-hoc Dunn’s test detected a significant difference in relative abundance between SMKN and all other sites (CYR: Z = −4.286, *p*_adj_ = 0.0001818; SBD: Z = 4.2018, *p*_adj_ = 0.0002648; SMKS: Z = 3.960, *p*_adj_ = 0.0007497; OWR: Z = 5.400, *p*_adj_ = 0.0000006674). No significant differences were found in pairwise comparisons of CYR, SBD, SMKS, and OWR.

The relative abundance of macrophytes increased with depth (Figure 5). Most taxa, however, did not display much variation in eDNA abundance with depth. Kruskal-Wallis test indicated that the difference in relative abundance between depths was not significant (*χ*^2^ = 0.61401, *df* = 2, *p* = 0.7356).

### Alpha diversity in sediment samples

Only sediment samples collected at SMKN, SMKS, and OWR contained any reads of identified MOTUs. Of the three sites, the sediment sample at SMKS had the highest taxon richness and diversity, followed by SMKN, and lastly OWR (Figure S6). However, SMKN had the highest cumulative relative abundance of macrophyte reads, followed by SMKS and lastly OWR (Figure S6).

### Environmental DNA community in water samples across season, site, and depth

PERMANOVA showed that eDNA MOTUs were found to be structured by season and site (Table 2; season: *df* = 2, *F* = 12.0268, *R*^2^ = 0.31995, *p* = 0.001; site: *df* = 4, *F* = 3.4534, *R*^2^ = 0.18374, *p* = 0.001). Further pairwise comparisons showed that differences were significant between the inter-monsoon and each monsoon, but not between two monsoons. Site-driven differences were significant only between CYR and OWR. No differentiation was found between depths.

**Table 2:** Summary of PERMANOVA test statistics for marine macroalgal eDNA detected among seasons, sites, and depths, based on the community data weighted by abundance

Variable	pseudo-*F*	*R*^2^	*p*-value
Season	12.027	0.320	0.001
Inter-monsoon vs. Southwest monsoon	13.207	0.321	0.003
Inter-monsoon vs. Northeast monsoon	13.488	0.325	0.003
Northeast monsoon vs. Southwest monsoon	2.713	0.088	0.144
Site	3.453	0.184	0.001
CYR vs. SBD	3.442	0.177	0.080
CYR vs. SMKN	2.662	0.143	0.610
CYR vs. SMKS	1.728	0.097	1.000
CYR vs. OWR	4.979	0.237	0.040
SBD vs. SMKN	1.701	0.096	1.000
SBD vs. SMKS	0.568	0.034	1.000
SBD vs. OWR	0.876	0.052	1.000
SMKN vs. SMKS	0.974	0.057	1.000
SMKN vs. OWR	3.226	0.168	0.560
SMKS vs. OWR	1.515	0.086	1.000
Depth	0.656	0.017	0.749
1 m vs. 10 m	0.375	0.013	0.853
1 m vs. 20 m	0.412	0.015	0.870
10 m vs. 20 m	0.057	0.012	0.876

**Figure 6:**
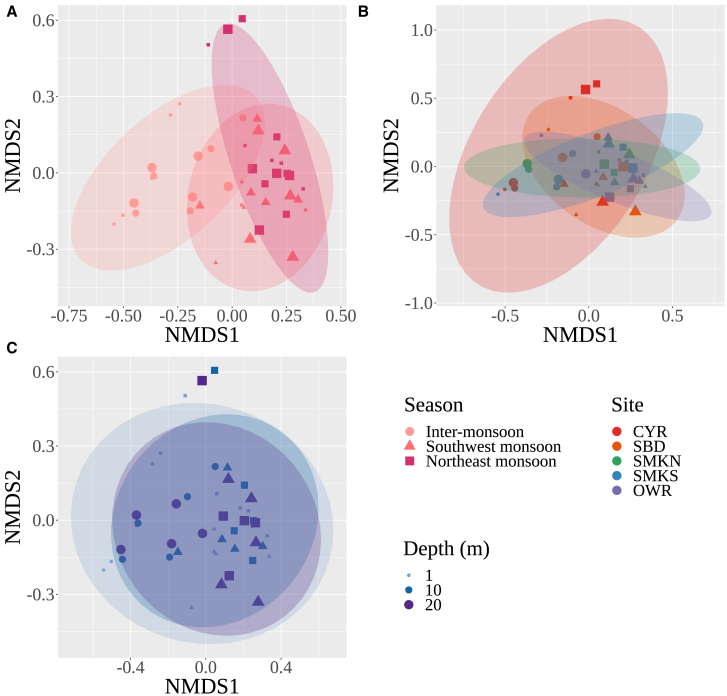
Nonmetric multidimensional scaling (nMDS) based on Bray-Curtis dissimilarity between sample communities sequenced for eDNA (stress = 0.14) (A) By season. (B) By site. (C) By depth.

The nMDS also showed that there were differences in macrophyte communities between the inter-monsoon and monsoons (Figure 6). Communities sampled during both monsoon seasons were mostly similar but distinct from those collected during the inter-monsoon season. Communities sampled at CYR—where seagrasses and macroalgae were located—were distinct from other sites, particularly during the northeast monsoon. During the monsoon season, communities at OWR were different from other sites. Conversely, communities at SMKN were distinct from other sites during the inter-monsoon season, partly due to those samples having the highest MOTU richness (78 in total). Communities at other sites were largely similar.

## Discussion

### Environmental DNA fingerprinting of tropical coastal macrophytes

Coastal macrophytes were detected in 50 of the 60 subtidal water and sediment samples from an area spanning 41.9 km^2^ in the Singapore Strait. eDNA metabarcoding successfully identified major lineages of macroalgae and was able to differentiate between red and brown macroalgae, even resolving their identities to genus for most of the 27 macrophyte taxa detected from 164,988 sequencing reads. The taxa include 19 identified families, of which 12 were previously found in Singapore and represent 24% of previously known families (Table S2). The four most common families were Sargassaceae, Peyssonneliaceae, Rhodomelaceae, and Ceramiaceae, each found in more than half of all samples. Sargassaceae is a brown macroalgal family that is widely distributed globally and includes genera well-represented in the tropics, such as *Sargassum* and *Turbinaria*, which have been locally reported to dominate the reef flat to the reef crest and grow on artificial structures.^63^^,^^64^^,^^65^ These taxa possess floating vesicles and may aggregate in surface rafts that disperse for long distances,^66^^,^^67^^,^^68^ thereby contributing significantly to eDNA reads even at the farthest site from shore. Peyssonneliaceae is a crustose red macroalgal family, with one genus (*Peysonnelia*) previously recorded in Singapore. Rhodomelaceae and Ceramiaceae are macroalgae under the diverse order Ceramiales.^69^ Rhodomelaceae is estimated to be the largest red macroalgal family globally, with 18 species previously recorded locally (Table S2), including in mangrove habitats.^70^ The low congruence between eDNA and traditional survey methods has been found in past studies, resulting from a number of potential reasons: incomplete reference libraries, false negatives stemming from single-marker assays, PCR inhibition and biases, and insufficient sequencing depth.^32^^,^^71^^,^^72^^,^^73^

Nonetheless, this study detected seven genera not previously recorded in Singapore, demonstrating added value in eDNA assessments of macrophyte diversity. Three of these genera—*Stylonema*, *Wrangelia*, and *Titanoderma*—have been found in Southeast Asia, i.e., Malaysia, Thailand, Vietnam, and the Philippines.^74^ While *Stylonema* was only detected in one sample and could be an anomaly, *Wrangelia* and *Titanoderma* were both found across all seasons and at least three sites each, raising the possibility that unknown populations of both genera exist. The other four genera—*Crustaphytum*, *Gloiocladia*, *Lomentaria*, and *Predaea*—are widely distributed,^75^ but were only detected in 1–2 samples. Further studies are required to confirm the presence of these genera in local macrophyte ecosystems.

The eDNA-unique detections—also reported in other studies—boost the potential of eDNA as a complementary, but not a substitutive, method to improve assessments of macrophyte diversity and potentially trace endemic macrophyte species back to blue carbon ecosystems, unlike coarser tracing methods.^18^^,^^27^^,^^73^ The cost-effectiveness and relatively low sampling effort of eDNA metabarcoding enable more frequent use, especially to study spatial and seasonal fluctuations that this study has demonstrated to significantly affect eDNA diversity and abundance.^76^^,^^77^^,^^78^

### Seasonal patterns of macrophyte environmental DNA

Our results showed that eDNA can detect significant variations in macroalgal richness, diversity, and relative abundance between monsoonal seasons, both within samples and on a community scale (especially between the inter-monsoon and both monsoon seasons). Comparisons of identified taxa showed a weak overlap between seasons at most sites, with identified taxa often found solely within 1–2 seasons, suggesting the strong seasonality of different macroalgal species (Figure 4). Additionally, *Sargassum* blooms and elevation of algal biomass occur at the start of the northeast monsoon, largely driven prior to that by higher seawater temperatures in the latter half of the year that boost metabolic and photosynthetic rates, thus initiating thalli growth.^56^^,^^64^ This increase in biomass is associated with greater eDNA abundance, leading to the surprisingly high macroalgal relative abundance in the northeast monsoon driven by Sargassaceae, which contributed to about half of all macroalgal sequencing reads (Figure 5).

eDNA may also be able to detect variations in macroalgal diversity associated with coastal development. Despite past surveys reporting high macroalgal richness at Cyrene Reefs,^79^ CYR was found to have relatively low richness and diversity with eDNA. This difference could be attributed to a decline in macrophyte diversity over time due to environmental changes, given its location in a busy channel, which has experienced significant coastal development.^79^^,^^80^^,^^81^^,^^82^

Critically, the relationship between eDNA read count and blue carbon biomass needs to be better quantified to translate changes in relative abundance to biomass for reliable blue carbon accounting. eDNA metabarcoding displays great promise in determining relative species abundance and biomass, but quantitative estimates of their relationship remain uncertain.^40^^,^^83^ This study was also unable to establish a strong relationship between eDNA read count and TOC due to little or inconsistent variation among sites and depths (Figure S3). A more predictive quantitative relationship needs to be established for more reliable eDNA estimations of spatial patterns and temporal changes in blue carbon. Apart from understanding the taxonomic origins of blue carbon (e.g., mangrove or seagrass species), sources of organic carbon in the water column or sediment could be further narrowed with stable isotopic measurements (δ^13^C and δ^15^N) or compound-specific isotope analysis (e.g., lipid and amino acid isotopes).^18^

### Spatial sampling of macrophyte environmental DNA for blue carbon monitoring

This study supports the need for an eDNA-based monitoring system to sample across spatial scales, given significant variations in macroalgal richness, diversity, and relative abundance between sites. Sites closer to known areas of high macroalgal species richness generally had higher eDNA richness and diversity—such as SMKN, SBD, and SMKS—with the farthest site, OWR, having the lowest. Additionally, only a weak overlap of identified taxa was found between sites.

Therefore, an eDNA monitoring system—especially one over a large area—would require a large spatial spread of sampling effort, given the higher site fidelity of eDNA in the tropics as demonstrated.^30^^,^^47^^,^^48^^,^^49^^,^^50^ This would inevitably raise the financial cost and manpower requirements for a monitoring system based on active eDNA sampling. However, monetary costs could be offset by the decreasing prices of molecular analysis and refinement of eDNA sampling techniques, including passive sampling approaches (e.g., submergence of filter membranes into the water column), which would reduce the labor needed for eDNA collection.^77^^,^^84^^,^^85^^,^^86^^,^^87^

Interestingly, there were no significant differences in eDNA diversity, richness, and abundance between different depths, suggesting that sampling across depths may not be necessary in similar subtidal ecosystems for blue carbon monitoring. This result corroborated findings from a previous eDNA study on local metazoan diversity.^27^ We posit that sampling depths were shallow and the Singapore Strait is vertically well-mixed throughout year,^88^ as opposed to contrasting results from other ecosystems with strong haloclines that reduce vertical mixing or were conducted over much larger depth ranges in the open ocean.^8^^,^^89^ Further sampling and confirmation are required, as our findings do contradict at least one study conducted in a dynamic coastal ecosystem which found that eDNA captured depth partitioning of fish diversity even at a maximum depth of 10 m, although it was conducted nearer to shore and in temperate waters.^90^

### Environmental constraints on macrophyte environmental DNA monitoring

Detection of coastal macrophyte eDNA appears to be weaker in equatorial waters as opposed to temperate regions or the deep sea. In particular, one-third of the sediment samples were not successfully PCR amplified. The low detection level of eDNA in sediment is surprising, given that eDNA amplification of marine macrophytes had been positive in several past studies.^34^^,^^91^ This limitation constrains the present study’s ability to characterize sediment-specific patterns and quantify the distribution of blue carbon at depth. While remineralization and mixing of organic carbon in the water column could curtail the export of detectable DNA to the seafloor,^8^ it is likely that the relatively short residence time of DNA in the warm, equatorial benthic environment here could limit levels of extractable eDNA.^92^^,^^93^^,^^94^ The type of sediment may also affect the affinity and binding of DNA to sediment and thus could influence eDNA persistence and detection limits.^95^ For instance, higher eDNA degradation rates were observed in soil with lower organic carbon content.^96^ Future studies focusing on comparing PCR amplification success among different sediment types, complemented by pre-processing of sediment such as decantation and homogenization,^97^ will help improve eDNA recovery from sediment samples.

Interestingly, fewer taxa were detected at sufficient read abundances for confident taxonomic identification than in temperate and deep waters, which have lower macrophyte diversity.^8^^,^^98^ Potentially, warmer waters at the equator result in shorter eDNA fragments that reduce PCR amplification success, possibly even more so than those of the far tropics, such as the Red Sea, where temperatures can be much lower during the winter.^25^^,^^99^^,^^100^^,^^101^

Fluctuations in environmental factors—such as rainfall and subtidal and residual flow velocity—may also potentially decrease eDNA detection. Macroalgal richness and diversity observed in this study were significantly lower in the northeast monsoon, which had higher local rainfall and flow velocity, corresponding with findings from past studies.^102^^,^^103^^,^^104^ Indeed, higher rainfall and subtidal flow velocity have been found to negatively impact eDNA detection by driving eDNA dispersion, homogenizing eDNA, and decreasing PCR success.^102^^,^^105^^,^^106^^,^^107^ This is a key consideration for any application of an eDNA-based monitoring system for blue carbon in the tropics, where global rainfall is concentrated.^108^ To account for this, future research should aim to establish a quantitative relationship between environmental factors and eDNA detection through methods such as numerical simulations and particle tracking that have shown potential for seagrass eDNA characterization.^109^^,^^110^

eDNA detection may also be negatively affected by rising sea temperatures arising from climate change. By 2100, global sea surface temperatures are set to increase by a median of 2.7°C (2.0°C–3.3°C) relative to 1995–2014 levels under business-as-usual scenarios.^111^ In Southeast Asia, the increase in median sea surface temperature is even higher at 2.8°C (2.3°C–3.5°C) by 2100, with the Singapore Strait already seeing an increase of nearly 1°C from just 2002 to 2019.^99^ Precipitation intensity has and is predicted to continue to increase significantly in the tropics.^112^^,^^113^^,^^114^ Both climatic changes have a negative impact on eDNA detection, potentially lowering the long-term efficacy of an eDNA-based monitoring system for blue carbon, especially in the tropics and warm, shallow-water ecosystems.^115^

### Conclusions

This study demonstrated that eDNA could be used effectively to detect and characterize tropical coastal macrophyte richness, diversity, and relative abundance, particularly for macroalgae. Significant variation was evident across seasons and sites, which must be accounted for by any future monitoring system in the region. Future studies should aim to improve the detection of mangrove, seagrass, and green macroalgal eDNA by designing more efficacious universal primers and creating a regional barcode reference library for macrophytes.

Apart from highlighting eDNA’s ability to provide insights into changes in coastal macrophyte communities, this study has also raised important considerations for a future monitoring system focusing on blue carbon in equatorial waters. Monsoonal variation is a key consideration for any eDNA research in the region, and sampling needs to be conducted at a wide variety of sites, though not necessarily at different depths. The higher degradation rate of eDNA in warmer waters also presents challenges in detection that require further investigation.

Blue carbon is a promising natural climate solution, but the associated ecosystems are currently under severe threat from anthropogenic impacts.^54^^,^^116^ Taxon-specific identification of blue carbon sources allows for more precise carbon accounting and prioritization of source habitats for conservation. More generally, in the species-rich tropics, eDNA is a cost- and time-effective approach to obtaining biodiversity data for informing blue carbon strategies.

### Limitations of the study

While at least trace levels of all three coastal macrophyte lineages were detected, sequences from some taxa ultimately did not meet conservative filtering requirements. For instance, mangrove sequences from the family Rhizophoraceae were found at CYR, but were removed as they were only detected in 1 of 5 PCR replicates (below our requirement of 3 of 5 replicates). Some other sequences were also removed as they were singletons. The successful detection of red and brown macroalgae is thus likely due in part to their relatively high abundance in local and regional waters and corresponding high export of macroalgal carbon.^8^^,^^56^^,^^117^ Conversely, the lower abundance and biomass of green macroalgae among sites in the Singapore Strait could have precluded eDNA detection.^56^ Despite the high biomass of living mangroves, the scarcity of mangrove sequences detected is possibly due to the lower organic carbon export from these habitats.^34^ This incongruity is evident even at Pulau Semakau, which hosts the largest mangrove forest in the vicinity of our sites.^54^

Apart from the limited biomass exported from benthic habitats, the lack of detection of seagrasses and green macroalgae taxa could also be due to primer bias by the universal Euka02 primers reported in past studies.^34^^,^^35^^,^^98^ Indeed, PCR can amplify eDNA from different species unevenly,^118^ and eDNA read counts, which have not been corrected for amplification biases, may not be predictive of species abundance or biomass.^119^ While the likelihood of seagrass primer biases may be low and the potential mechanisms of such biases unclear, green macroalgae may have a high incidence of introns, reducing the ability of primers to anneal to the DNA template precisely.^35^ Therefore, designing better-matched universal primers could help improve the detection of coastal macrophytes. Experiments to understand issues with the detection of mangrove and seagrass eDNA in tropical waters should also be conducted, given their importance as blue carbon ecosystems. Past analyses of mangrove and seagrass contributions to allochthonous carbon have shown mixed results, but their inputs are generally lower than those of macroalgae.^10^^,^^15^^,^^34^ This potentially explains the discrepancy of past *in-situ* detection of mangroves and seagrasses with our study, which sampled areas between ecosystems. Experiments on the residence time of DNA in tropical waters would provide a more precise model of where and when eDNA could be found in the water.^120^

Importantly, taxonomic assignment of eDNA reads is limited by the availability and accuracy of reference DNA in the database.^35^^,^^121^ Past studies have found significant improvements in identification success with the inclusion of a country- or region-specific barcode reference database.^34^^,^^100^^,^^122^^,^^123^ While this study did not have access to a barcode reference library for the Singapore Strait, the compilation of one that builds on existing work in this region could significantly increase the number of taxa identified, given that 97.4% of sequences remained unclassified when matched against macrophyte references from NCBI.^32^^,^^59^^,^^65^^,^^124^^,^^125^ Indeed, if local genotypes are compiled into a regional or global database over time, the long-term benefits of such a resource would extend to fields beyond blue carbon.

## Resource availability

### Lead contact

Further information and requests for resources should be directed to and will be fulfilled by the lead contact, Danwei Huang (huangdanwei@nus.edu.sg).

### Materials availability

This study did not generate new unique reagents.

### Data and code availability

• Data: All sequencing data supporting this study are publicly available at NCBI Sequence Read Archive (SRA) under BioProject: PRJNA1090548.
• Code: Demultiplexing information and a custom Kraken 2 classification database are publicly available at https://doi.org/10.5281/zenodo.17141230.
• Any additional information required to reanalyze the data reported in this work is available from the lead contact upon request.

## Acknowledgments

We thank Yin Cheong Aden Ip, Matthew Hui-Chieh Ng, Zhi Ting Yip, and Wan Wen Rochelle Chan of the Reef Ecology Laboratory, the 10.13039/501100001352National University of Singapore, for field and laboratory support. This study was supported by the 10.13039/501100001321National Research Foundation, Prime Minister’s Office, Singapore, under the 2025 Southeast Asian Deep Sea Biodiversity Expedition (A-8000658-00-00), the International SeaKeepers Society, and Temasek (A-0008400-02-00). We acknowledge the St John’s Island National Marine Laboratory (SJINML) for providing R/V *Galaxea* necessary for conducting the research. SJINML is a National Research Infrastructure under the National Research Foundation Singapore. We are also grateful to Elvagris Segovia Estrada for assistance with sediment TOC analysis. All necessary sampling and surveys were authorized by the National Parks Board, Singapore (NP/RP22-039).

## Author contributions

Conceptualization, W.J.D.T., J.J.M.C., V.K., and D.H.; methodology, W.J.D.T., J.J.M.C., and V.K.; investigation, W.J.D.T., J.J.M.C., and V.K.; formal analysis, W.J.D.T. and J.J.M.C.; writing – original draft, W.J.D.T.; writing – review and editing, J.J.M.C., V.K., and D.H.; supervision, D.H.; and funding acquisition, D.H.

## Declaration of interests

The authors declare that they have no conflict of interest.

## STAR★Methods

### Key resources table

**Table:** 

REAGENT or RESOURCE	SOURCE	IDENTIFIER
**Biological samples**		

Water samples	This study	See Table S1
Sediment samples	This study	See Table S1

**Critical commercial assays**		

GoTaq G2 Master Mix (Green)	Promega	M7823
NEBNext Ultra II DNA Library Prep Kit for Illumina	New England BioLabs	E7645S
Illumina TruSeq DNA CD Indexes	Illumina	20015949

**Deposited data**		

Raw sequence reads	This study	NCBI BioProject: PRJNA1090548
Demultiplexing information	This study	Zenodo: https://doi.org/10.5281/zenodo.17141230
Custom Kraken 2 classification database	This study	Zenodo: https://doi.org/10.5281/zenodo.17141230

**Oligonucleotides**		

Euka02 primers to amplify 18S-V7 rRNA Forward: 5’-TTTGTCTGSTTAATTSCG-3’ Reverse: 5’-CACACAGACCTGTTATTGC-3’	Guardiola et al.^126^	N/A

**Software and algorithms**		

PEAR v0.9.6	Zhang et al.^127^	https://cme.h-its.org/exelixis/web/software/pear/doc.html
OBITools v1.2.13	Boyer et al.^128^	https://pythonhosted.org/OBITools/welcome.html
CRABS v0.1.1	Jeunen et al.^129^	https://github.com/gjeunen/reference_database_creator
Kraken v2.1.2	Wood et al.^130^	https://github.com/DerrickWood/kraken2
NCBI BLAST+ v2.13.0	Camacho et al.^131^	https://ftp.ncbi.nlm.nih.gov/blast/executables/blast+/
MAFFT v7.5.11	Katoh and Standley^132^	https://mafft.cbrc.jp/alignment/software/
Objective Clustering	Meier et al.^133^^,^^134^	https://github.com/asrivathsan/obj_cluster
R v4.2.2	R Core Team^135^	https://www.r-project.org/
vegan v2.6-4	Oksanen et al.^136^	https://cran.r-project.org/web/packages/vegan/index.html
pairwiseAdonis v0.4	Martinez Arbizu^137^	https://github.com/pmartinezarbizu/pairwiseAdonis

### Method details

#### Sample collection

To characterise seasonal changes in blue carbon eDNA, water and sediment samples were collected during daytime from April to December 2022 in three sampling campaigns—once during the inter-monsoon period and once during each of the two monsoon seasons (Table S1). Total organic carbon (TOC) analysis was also performed for water and sediment samples collected in the monsoon periods to test for relationships of TOC between sample types and with macrophyte eDNA read abundance. Collections were conducted at five offshore localities in the Singapore Strait (Figure 1), with four of the five chosen for their proximity to areas of high coastal macrophyte richness. Pulau Subar Darat (SBD) and Cyrene Reefs (CYR) are known to have high *Sargassum* cover.^56^^,^^64^ CYR and Pulau Semakau (SMKN and SMKS) host the largest seagrass meadows in Singapore.^60^ Pulau Semakau also has natural and replanted mangroves along almost the entirety of its perimeter.^138^^,^^139^ The last site (OWR) is in an open water environment ∼3 km south of Pulau Semakau, away from areas of high coastal macrophyte richness.

Water samples were taken from different depths (1 m, 10 m, 20 m) at each site using a 5 L Van Dorn horizontal point water sampler (WaterMark). For eDNA analysis, a 1 L sample was transferred to a sterilised bottle. For TOC analysis, a 50-ml sample was transferred to a sterile falcon tube. All samples were kept on ice on board the research vessel and during transport to the lab. Water samples for eDNA analysis were vacuum filtered in the lab (1 L per filter), using sterile nylon filter membranes (ThermoFisher Scientific; diameter, 47 mm, pore size, 0.22 μm). Filter membranes and water samples (for TOC analysis) were stored at -80°C until further processing.

Sediment samples were collected from the surface (top 1 cm) of the seabed at each site using a Van Veen grab sampler. For both eDNA and TOC analysis, samples were collected using sterile 50-ml falcon tubes. Open tubes were inserted into the sediment surface and lifted out with the sediment sample and sealed. All sediment samples were handled similarly to water samples as described above.

#### DNA extraction, genotyping, and library preparation

Intracellular and extracellular eDNA from the filter membrane was extracted following Ip et al.^27^^,^^28^ Filter membranes were cut up and split into two subsamples. Each subsample was incubated for 3 h at 55°C in 20 μl of 20 mg/ml proteinase K and 900 μl CTAB (hexadecyltrimethylammonium bromide) buffer. The digest was subsequently purified via phase separation using phenol:chloroform:isoamyl-alcohol (25:24:1) and incubated for 16 h at -20°C in 60% isopropanol to increase eDNA recovery and yield. eDNA pellets were resuspended in 70 μl of nuclease-free water. Subsamples from the same sample were combined and stored at -20°C.

eDNA from each sample was amplified in five polymerase chain reaction (PCR) replicates of 25 μl by adding 1 μl of bovine serum albumin (New England Biolabs), 1 μl of magnesium chloride (New England Biolabs), 12.5 μl of GoTaq Green Master Mix (Promega), 1 μl each of 5 μM tagged Euka02 primers (F- 5’-TTTGTCTGSTTAATTSCG-3’, R- 5’-CACAGACCTGTTATTGC-3’,^126^ 5 μl of eDNA template, and 3.5 μl of nuclease-free water. PCR primers were dual-indexed with each pair tagged with the same unique 8-bp barcode at the 5′ end to enable sample demultiplexing. The thermocycling profile was 15 min at 95°C, 35 cycles of 30 s at 94°C, 45 s at 55°C, 1.5 min at 72°C, and lastly 10 min at 72°C. The Euka02 primers target a short, hypervariable fragment (100–110 bp) of nuclear ribosomal 18S rRNA gene in the v7 region and have demonstrated the ability to amplify a broad range of macrophytes and accurately identify 71% of sequences down to genus level.^35^^,^^126^ Amplification success was checked using gel electrophoresis.

A total of 250 PCR products and 160 negative controls were aggregated into six pools. Amplicons were purified using 1.8× AMPure XP (Beckman Coulter) bead-to-sample ratio and six libraries were prepared using the NEBNext Ultra II DNA Library Prep Kit for Illumina (New England Biolabs) multiplexed with Illumina TruSeq CD indexes. These were pooled and sequenced on three HiSeqX lanes (150 bp, paired-end) at NovogeneAIT Genomics Singapore Pte Ltd. Each lane was spiked with 20% PhiX to improve base diversity.

Contamination control measures were strictly administered for all field and laboratory processes. The water sampler was rinsed with detergent and autoclaved ultra-pure water between collections at different sites. Field negative controls were also collected at every site by rinsing the water sampler with 1 L of autoclaved ultra-pure water which was dispensed into a sterile bottle. Collection bottles, falcon tubes and filtration equipment (filter units and membranes) were autoclaved before use. DNA extractions and PCR preparations were conducted in a dedicated biological safety cabinet which was cleaned with 10% household bleach and 70% ethanol and UV-sterilised before use. PCR negative controls were also run. Where possible, instruments and consumables were autoclaved, and all surfaces (including storage boxes) and instruments were frequently cleaned with both 10% bleach and 70% ethanol and UV-sterilised before use.

#### Bioinformatics

FASTQ files were processed following the protocol of Ip et al.^27^ Illumina paired-end sequencing reads were merged using PEAR v0.9.6.^127^ The following parameters were used: minimum assembly length (n) = 100, maximum assembly length (m) = 300, quality score threshold (q) = 30, and minimum read length post-trim (t) = 110. OBITools v1.2.13^128^ was used in demultiplexing and other downstream processing of assembled reads. *ngsfilter* was utilised with default settings to demultiplex sequence reads to respective PCR replicates using both primer and library tags. *obiuniq* was then used to group sequence records and remove duplicates across libraries.

CRABS v0.1.1^129^ was used to create a custom database containing sequences downloaded using *db_download* from the NCBI *nt* database (accessed 6 February 2023) with a list of mangrove, seagrass and macroalgal taxa (Table S3) and ‘rRNA’ as search terms. *db_merge* was used to merge the downloaded files and remove any duplicates, resulting in a total of 30,625 sequences. Using the downloaded sequences from CRABS, *kraken2-build* from Kraken v2.1.2^130^ was used to build a Kraken 2 database. *kraken2* was then used to classify all reads and assign each read a taxonomic identity. Six different scores—0, 0.1, 0.3, 0.5, 0.7, and 0.9—were tested for their classification success rate (Table S4) and accuracy when compared to classification with *blastn* implemented with BLAST+ v2.13.0.^131^ Scores of 0.7 and 0.9 saw poor classification success at only 1.05% and 0.18% of reads classified respectively. While scores of 0.1 and 0.3 showed higher classification rates, false positives were found. For suitable balance between classification success and accuracy, a score of 0.5 was selected for classification.^140^^,^^141^

Reads were distributed to replicates using *obisubset* and read counts for unique sequences in each replicate were obtained with *obistat*. Using *obigrep*, a length filter was applied to retain only reads between 90 and 110 bp in length.^126^ Sequences with read counts of <2 were also removed. *obiclean* was then used to identify any potential sequencing errors by selecting only head and singleton reads. *obistat* was used to extract the number of reads per unique sequence for each PCR replicate. Only sequences present in at least three of the total five PCR replicates per sample were subsequently retained and pooled into their respective samples with a combined read count per sequence. These steps eliminated rare reads which likely contained amplification and sequencing errors. Sequences found in negative controls (see Table S1) were deemed contaminants and removed before downstream analyses. Remaining sequences across all samples were concatenated into a single dataset and aligned with MAFFT v.7.5.11 using default options.^132^ Objective clustering was employed to delimit putative species units by grouping sequence reads into molecular operational taxonomic units (MOTUs).^133^^,^^134^ A threshold of 4% difference was used to provide a conservative estimate of genus-level diversity for extracellular DNA based on multiple studies using different regions (v1-v2 and v7-v8) of the 18S rRNA gene.^126^

Lastly, *blastn* implemented with BLAST+ v2.13.0^131^ was used to match sequences against the NCBI *nt* database (downloaded 15 February 2023) (≥80% sequence similarity and e-value ≤10^-6^). Genus-level identification was assigned only if sequence matches met a ≥96% percentage identity threshold. Otherwise, sequence matches between <96% and ≥80% were only associated with the closest available taxon. Sequences were also searched using *blastn* against the SILVA 138.2 SSU Ref NR 99 database^142^^,^^143^ (downloaded 18 July 2025) to validate our results based on the NCBI *nt* database. A list of locally-recorded mangrove, seagrass and macroalgal species was compiled from previous field surveys for comparison with identified MOTUs.^59^^,^^60^^,^^74^^,^^139^^,^^144^^,^^145^^,^^146^^,^^147^

#### Total organic carbon analysis

TOC analysis was conducted at Marchwood Laboratory Services. Water samples were first homogenised and diluted as necessary before high-temperature catalytic oxidation. All bicarbonate and carbonate ions were converted to CO_2_ by acidification and purged out of the sample. A small amount of each sample was vaporised in a heated reaction chamber filled with a platinum catalyst, and CO_2_ produced from the oxidation of organic carbon was measured by non-dispersive infrared detection.

Sediment samples were oven dried for 144 h at 60°C, ground and homogenised for TOC estimation using the dry combustion method.^148^ A subsample (10–30 mg) was placed in a silver-foil boat (6 × 6 × 12 mm) and mixed with distilled water (50 μl) before being placed in a vacuum desiccator and acid-fumigated for 6 h in 100 ml of 12 mol L^-1^ hydrochloric (HCl) to remove inorganic carbonate materials.^149^ Subsequently, samples were oven dried for 4 h at 60°C to remove excess acid before being weighed. The TOC content of each sediment sample was determined using an Elementar vario TOC cube carbon analyser through catalytic oxidation at 950°C.

### Quantification and statistical analysis

All statistical analyses were conducted in R v4.2.2.^135^ Samples were grouped categorically by depth (1 m, 10 m, and 20 m), site (CYR, SBD, SMKN, SMKS, OWR), and season (inter-monsoon, southwest monsoon, and northeast monsoon) to compare blue carbon community structure. To analyse alpha diversity—the diversity of MOTUs in each water sample—taxon richness and the Shannon diversity index were calculated for each sample. Kruskal-Wallis test and post-hoc Dunn’s tests with Bonferroni correction were conducted to examine relationships between taxon richness and Shannon diversity with depth, site, and season.

Analyses of community structure were conducted using the *vegan* v2.6-4 package.^136^ MOTU read counts were transformed into relative abundance using *decostand* to avoid suboptimal or erroneous eDNA variance.^150^ Differences in macrophyte assemblages across sites, depths and seasons were evaluated using the Bray-Curtis dissimilarity index and visualised on a nonmetric multidimensional scaling (nMDS) plot. Homogeneity of dispersion was verified with *betadisper* before performing a permutational multivariate analysis of variance (PERMANOVA) using *adonis2* to test whether MOTU compositional differences were associated with site, depth, and/or seasonality. Pairwise PERMANOVA tests were conducted with *pairwiseAdonis* v0.4 custom script (https://github.com/pmartinezarbizu/pairwiseAdonis)^137^ with Bonferroni correction applied for multiple testing. A principal component analysis (PCA) was also conducted with relative abundance and Hellinger transformation of MOTU read counts using *decostand* and *rda,* respectively, and visualised with *biplot* and *ordiellipse*.

## Supplemental information

Document S1. Figures S1–S6

Table S1. Sampling data, including locality information, collection date and time, as well as sample type and volume

Table S2. Compiled database of macroalgal species in Singapore based on previous studies

Table S3. Mangrove, seagrass, and macroalgal taxon keywords used to generate the custom database of sequences

Table S4. Kraken 2 confidence scores and corresponding percentages of sequences successfully assigned a taxonomic identity

## References

[1] Chausson A., Turner B., Seddon D., Chabaneix N., Girardin C.A.J., Kapos V., Key I., Roe D., Smith A., Woroniecki S. (2020). Mapping the effectiveness of nature-based solutions for climate change adaptation. Glob. Chang. Biol..

[2] Nesshöver C., Assmuth T., Irvine K.N., Rusch G.M., Waylen K.A., Delbaere B., Haase D., Jones-Walters L., Keune H., Kovacs E. (2017). The science, policy and practice of nature-based solutions: An interdisciplinary perspective. Sci. Total Environ..

[3] Lovelock C.E., Duarte C.M. (2019). Dimensions of Blue Carbon and emerging perspectives. Biol. Lett..

[4] Donato D.C., Kauffman J.B., Murdiyarso D., Kurnianto S., Stidham M., Kanninen M. (2011). Mangroves among the most carbon-rich forests in the tropics. Nat. Geosci..

[5] Fourqurean J.W., Duarte C.M., Kennedy H., Marbà N., Holmer M., Mateo M.A., Apostolaki E.T., Kendrick G.A., Krause-Jensen D., McGlathery K.J. (2012). Seagrass ecosystems as a globally significant carbon stock. Nat. Geosci..

[6] Trevathan-Tackett S.M., Kelleway J., Macreadie P.I., Beardall J., Ralph P., Bellgrove A. (2015). Comparison of marine macrophytes for their contributions to blue carbon sequestration. Ecology.

[7] Krause-Jensen D., Lavery P., Serrano O., Marbà N., Masque P., Duarte C.M. (2018). Sequestration of macroalgal carbon: the elephant in the Blue Carbon room. Biol. Lett..

[8] Ortega A., Geraldi N.R., Alam I., Kamau A.A., Acinas S.G., Logares R., Gasol J.M., Massana R., Krause-Jensen D., Duarte C.M. (2019). Important contribution of macroalgae to oceanic carbon sequestration. Nat. Geosci..

[9] Krause-Jensen D., Duarte C.M. (2016). Substantial role of macroalgae in marine carbon sequestration. Nat. Geosci..

[10] Macreadie P.I., Anton A., Raven J.A., Beaumont N., Connolly R.M., Friess D.A., Kelleway J.J., Kennedy H., Kuwae T., Lavery P.S. (2019). The future of Blue Carbon science. Nat. Commun..

[11] Sun Y., Mao S., Wang F., Peng P., Chai P. (2013). Identification of the Kukersite-type source rocks in the Ordovician Stratigraphy from the Tarim Basin, NW China. Chin. Sci. Bull..

[12] Xie X., Volkman J.K., Qin J., Borjigin T., Bian L., Zhen L. (2014). Petrology and hydrocarbon potential of microalgal and macroalgal dominated oil shales from the Eocene Huadian Formation, NE China. Int. J. Coal Geol..

[13] Zhang S., Zhang B., Bian L., Jin Z., Wang D., Chen J. (2007). The Xiamaling oil shale generated through Rhodophyta over 800 Ma ago. Sci. China Ser. D..

[14] Hardison A.K., Canuel E.A., Anderson I.C., Veuger B. (2010). Fate of macroalgae in benthic systems: carbon and nitrogen cycling within the microbial community. Mar. Ecol. Prog. Ser..

[15] Hidayah N., Ng C.T., Arina N., Fairoz M., Rozaimi M. (2022). Macroalgal and mangrove provenances demonstrate their relevance in contributing to the blue carbon pool of a tropical seagrass meadow. Ecol. Res..

[16] Smale D.A., Moore P.J., Queirós A.M., Higgs N.D., Burrows M.T. (2018). Appreciating interconnectivity between habitats is key to blue carbon management. Front. Ecol. Environ..

[17] Hyndes G.A., Nagelkerken I., McLeod R.J., Connolly R.M., Lavery P.S., Vanderklift M.A. (2014). Mechanisms and ecological role of carbon transfer within coastal seascapes. Biol. Rev..

[18] Geraldi N.R., Ortega A., Serrano O., Macreadie P.I., Lovelock C.E., Krause-Jensen D., Kennedy H., Lavery P.S., Pace M.L., Kaal J., Duarte C.M. (2019). Fingerprinting blue carbon: rationale and tools to determine the source of organic carbon in marine depositional environments. Front. Mar. Sci..

[19] Greiner J.T., Wilkinson G.M., McGlathery K.J., Emery K.A. (2016). Sources of sediment carbon sequestered in restored seagrass meadows. Mar. Ecol. Prog. Ser..

[20] Kennedy H., Beggins J., Duarte C.M., Fourqurean J.W., Holmer M., Marbà N., Middelburg J.J. (2010). Seagrass sediments as a global carbon sink: Isotopic constraints. Glob. Biogeochem. Cycles.

[21] Gillis L.G., Ziegler A.D., van Oevelen D., Cathalot C., Herman P.M.J., Wolters J.W., Bouma T.J. (2014). Tiny is mighty: seagrass beds have a large role in the export of organic material in the tropical coastal zone. PLoS One.

[22] Dubois S., Savoye N., Grémare A., Plus M., Charlier K., Beltoise A., Blanchet H. (2012). Origin and composition of sediment organic matter in a coastal semi-enclosed ecosystem: An elemental and isotopic study at the ecosystem space scale. J. Mar. Syst..

[23] Hill R., Bellgrove A., Macreadie P.I., Petrou K., Beardall J., Steven A., Ralph P.J. (2015). Can macroalgae contribute to blue carbon? An Australian perspective. Limnol. Oceanogr..

[24] Wesselmann M., Geraldi N.R., Duarte C.M., Garcia-Orellana J., Díaz-Rúa R., Arias-Ortiz A., Hendriks I.E., Apostolaki E.T., Marbà N. (2021). Seagrass (*Halophila stipulacea*) invasion enhances carbon sequestration in the Mediterranean Sea. Glob. Chang. Biol..

[25] Ruppert K.M., Kline R.J., Rahman M.S. (2019). Past, present, and future perspectives of environmental DNA (eDNA) metabarcoding: A systematic review in methods, monitoring, and applications of global eDNA. Glob. Ecol. Conserv..

[26] Taberlet P., Coissac E., Hajibabaei M., Rieseberg L.H. (2012). Environmental DNA. Mol. Ecol..

[27] Ip Y.C.A., Tay Y.C., Chang J.J.M., Ang H.P., Tun K.P.P., Chou L.M., Huang D., Meier R. (2021). Seeking life in sedimented waters: Environmental DNA from diverse habitat types reveals ecologically significant species in a tropical marine environment. Environ. DNA.

[28] Ip Y.C.A., Chang J.J.M., Lim K.K.P., Jaafar Z., Wainwright B.J., Huang D. (2021). Seeing through sedimented waters: environmental DNA reduces the phantom diversity of sharks and rays in turbid marine habitats. BMC Ecol. Evol..

[29] Rees H.C., Maddison B.C., Middleditch D.J., Patmore J.R.M., Gough K.C. (2014). The detection of aquatic animal species using environmental DNA – a review of eDNA as a survey tool in ecology. J. Appl. Ecol..

[30] Thomsen P.F., Willerslev E. (2015). Environmental DNA – An emerging tool in conservation for monitoring past and present biodiversity. Biol. Conserv..

[31] Bunholi I.V., Foster N.R., Casey J.M. (2023). Environmental DNA and RNA in aquatic community ecology: Toward methodological standardization. Environ. DNA.

[32] Ip Y.C.A., Chang J.J.M., Oh R.M., Quek Z.B.R., Chan Y.K.S., Bauman A.G., Huang D. (2023). Seq’ and ARMS shall find: DNA (meta)barcoding of Autonomous Reef Monitoring Structures across the tree of life uncovers hidden cryptobiome of tropical urban coral reefs. Mol. Ecol..

[33] Foster N.R., Jones A.R., Serrano O., Lafratta A., Lavery P.S., van Dijk K.j., Biffin E., Gillanders B.M., Young J., Masque P. (2024). Environmental DNA identifies coastal plant community shift 1,000 years ago in Torrens Island, South Australia. Commun. Earth Environ..

[34] Ortega A., Geraldi N.R., Duarte C.M. (2020). Environmental DNA identifies marine macrophyte contributions to Blue Carbon sediments. Limnol. Oceanogr..

[35] Ortega A., Geraldi N.R., Díaz-Rúa R., Ørberg S.B., Wesselmann M., Krause-Jensen D., Duarte C.M. (2020). A DNA mini-barcode for marine macrophytes. Mol. Ecol. Resour..

[36] Landenmark H.K.E., Forgan D.H., Cockell C.S. (2015). An estimate of the total DNA in the biosphere. PLoS Biol..

[37] Doi H., Uchii K., Takahara T., Matsuhashi S., Yamanaka H., Minamoto T. (2015). Use of droplet digital PCR for estimation of fish abundance and biomass in environmental DNA surveys. PLoS One.

[38] Doi H., Inui R., Akamatsu Y., Kanno K., Yamanaka H., Takahara T., Minamoto T. (2017). Environmental DNA analysis for estimating the abundance and biomass of stream fish. Freshw. Biol..

[39] Lacoursière-Roussel A., Côté G., Leclerc V., Bernatchez L. (2016). Quantifying relative fish abundance with eDNA: a promising tool for fisheries management. J. Appl. Ecol..

[40] Lamb P.D., Hunter E., Pinnegar J.K., Creer S., Davies R.G., Taylor M.I. (2019). How quantitative is metabarcoding: A meta-analytical approach. Mol. Ecol..

[41] Lodge D.M., Turner C.R., Jerde C.L., Barnes M.A., Chadderton L., Egan S.P., Feder J.L., Mahon A.R., Pfrender M.E. (2012). Conservation in a cup of water: estimating biodiversity and population abundance from environmental DNA. Mol. Ecol..

[42] Pilliod D.S., Goldberg C.S., Arkle R.S., Waits L.P. (2013). Estimating occupancy and abundance of stream amphibians using environmental DNA from filtered water samples. Can. J. Fish. Aquat. Sci..

[43] Shelton A.O., Kelly R.P., O’Donnell J.L., Park L., Schwenke P., Greene C., Henderson R.A., Beamer E.M. (2019). Environmental DNA provides quantitative estimates of a threatened salmon species. Biol. Conserv..

[44] Takahara T., Minamoto T., Yamanaka H., Doi H., Kawabata Z. (2012). Estimation of fish biomass using environmental DNA. PLoS One.

[45] Van Driessche C., Everts T., Neyrinck S., Halfmaerten D., Haegeman A., Ruttink T., Bonte D., Brys R. (2023). Using environmental DNA metabarcoding to monitor fish communities in small rivers and large brooks: Insights on the spatial scale of information. Environ. Res..

[46] Yamamoto S., Minami K., Fukaya K., Takahashi K., Sawada H., Murakami H., Tsuji S., Hashizume H., Kubonaga S., Horiuchi T. (2016). Environmental DNA as a ‘snapshot’ of fish distribution: a case study of Japanese jack mackerel in Maizuru Bay, Sea of Japan. PLoS One.

[47] Collins R.A., Wangensteen O.S., O’Gorman E.J., Mariani S., Sims D.W., Genner M.J. (2018). Persistence of environmental DNA in marine systems. Commun. Biol..

[48] Murakami H., Yoon S., Kasai A., Minamoto T., Yamamoto S., Sakata M.K., Horiuchi T., Sawada H., Kondoh M., Yamashita Y. (2019). Dispersion and degradation of environmental DNA from caged fish in a marine environment. Fish. Sci..

[49] Joseph C., Faiq M.E., Li Z., Chen G. (2022). Persistence and degradation dynamics of eDNA affected by environmental factors in aquatic ecosystems. Hydrobiologia.

[50] Strickler K.M., Fremier A.K., Goldberg C.S. (2015). Quantifying effects of UV-B, temperature, and pH on eDNA degradation in aquatic microcosms. Biol. Conserv..

[51] Hansen B.K., Jacobsen M.W., Middelboe A.L., Preston C.M., Marin R., Bekkevold D., Knudsen S.W., Møller P.R., Nielsen E.E. (2020). Remote, autonomous real-time monitoring of environmental DNA from commercial fish. Sci. Rep..

[52] Andruszkiewicz Allan E., Zhang W.G., C Lavery A., F Govindarajan A. (2021). Environmental DNA shedding and decay rates from diverse animal forms and thermal regimes. Environ. DNA.

[53] Székely D., Corfixen N.L., Mørch L.L., Knudsen S.W., McCarthy M.L., Teilmann J., -Jørgensen M.P., Olsen M.T. (2021). Environmental DNA captures the genetic diversity of bowhead whales *Balaena mysticetus* in West Greenland. Environ. DNA.

[54] Friess D.A., Gatt Y.M., Fung T.K., Alemu J.B., Bhatia N., Case R., Chua S.C., Huang D., Kwan V., Lim K.E. (2023). Blue carbon science, management and policy across a tropical urban landscape. Landsc. Urban Plan..

[55] Nguyen N.T.H., Friess D.A., Todd P.A., Mazor T., Lovelock C.E., Lowe R., Gilmour J., Ming Chou L., Bhatia N., Jaafar Z. (2022). Maximising resilience to sea-level rise in urban coastal ecosystems through systematic conservation planning. Landsc. Urban Plan..

[56] Kwan V., Fong J., Ng C.S.L., Huang D. (2022). Temporal and spatial dynamics of tropical macroalgal contributions to blue carbon. Sci. Total Environ..

[57] Lai S., Tan J., Deng M., Tan C., Thinesh K. (2022). A Guide to Implementing Coastal Nature-Based Solutions for Singapore (Centre for Liveable Cities).

[58] Tan Y.H.J., Tham J.K.Q., Paul A., Rana U., Ang H.P., Nguyen N.T.H., Yee A.T.K., Leong B.P.I., Drummond S., Tun K.P.P. (2023). Remote sensing mapping of the regeneration of coastal natural habitats in Singapore: Implications for marine conservation in tropical cities. Singap. J. Trop. Geogr..

[59] Kwan V., Shantti P., Lum E.Y.Y., Ow Y.X., Huang D. (2023). Diversity and phylogeny of seagrasses in Singapore. Aquat. Bot..

[60] Yaakub S.M., Lim R.L.F., Lim W.L., Todd P.A. (2013). The diversity and distribution of seagrass in Singapore. Nature in Singapore.

[61] Yang S., Lim R.L.F., Sheue C.-R., Yong J.W.H. (2013). Proceedings of Nature Society, Singapore’s Conference on ‘Nature Conservation for a Sustainable Singapore.

[62] Kerswell A.P. (2006). Global biodiversity patterns of benthic marine algae. Ecology.

[63] Low J.K.Y., Chou L.M., Phang S.-M., Lim P.E. (2013). Institute of Ocean and Earth Sciences.

[64] Low J.K.Y., Fong J., Todd P.A., Chou L.M., Bauman A.G. (2019). Seasonal variation of *Sargassum ilicifolium* (Phaeophyceae) growth on equatorial coral reefs. J. Phycol..

[65] Yip Z.T., Quek Z.B.R., Low J.K.Y., Wilson B., Bauman A.G., Chou L.M., Todd P.A., Huang D. (2018). Diversity and phylogeny of *Sargassum* (Fucales, Phaeophyceae) in Singapore. Phytotaxa.

[66] Andréfouët S., Zubia M., Payri C. (2004). Mapping and biomass estimation of the invasive brown algae *Turbinaria ornata* (Turner) J. Agardh and *Sargassum mangarevense* (Grunow) Setchell on heterogeneous Tahitian coral reefs using 4-meter resolution IKONOS satellite data. Coral Reefs.

[67] van Hees D.H., Olsen Y.S., Mattio L., Ruiz-Montoya L., Wernberg T., Kendrick G.A. (2019). Cast adrift: Physiology and dispersal of benthic *Sargassum spinuligerum* in surface rafts. Limnol. Oceanogr..

[68] Zubia M., Stiger-Pouvreau V., Mattio L., Payri C.E., Stewart H.L. (2020). A comprehensive review of the brown macroalgal genus *Turbinaria* J.V. Lamouroux (Fucales, Sargassaceae). J. Appl. Phycol..

[69] Díaz-Tapia P., Pasella M.M., Verbruggen H., Maggs C.A. (2019). Morphological evolution and classification of the red algal order Ceramiales inferred using plastid phylogenomics. Mol. Phylogenet. Evol..

[70] West J.A. (1991). New algal records from the Singapore mangroves. Gardens’ Bulletin Singapore.

[71] Ip Y.C.A., Chang J.J.M., Tun K.P.P., Meier R., Huang D. (2023). Multispecies environmental DNA metabarcoding sheds light on annual coral spawning events. Mol. Ecol..

[72] Ip Y.C.A., Chang J.J.M., Huang D. (2023). Advancing and integrating “Biomonitoring 2.0” with new molecular tools for marine biodiversity and ecosystem assessments. Oceanogr. Mar. Biol. Annu. Rev..

[73] McElroy M.E., Dressler T.L., Titcomb G.C., Wilson E.A., Deiner K., Dudley T.L., Eliason E.J., Evans N.T., Gaines S.D., Lafferty K.D. (2020). Calibrating environmental DNA metabarcoding to conventional surveys for measuring fish species richness. Front. Ecol. Evol..

[74] Phang S.-M., Yeong H.-Y., Ganzon-Fortes E.T., Lewmanomont K., Prathep A., Hau L.N., Gerung G.S., Tan K.S. (2016). Marine algae of the South China Sea bordered by Indonesia, Malaysia, Philippines, Singapore, Thailand and Vietnam. Raffles Bull. Zool..

[75] Guiry M.D., Guiry G.M. (2023). AlgaeBase. https://www.algaebase.org.

[76] Akre T.S., Parker L.D., Ruther E., Maldonado J.E., Lemmon L., McInerney N.R. (2019). Concurrent visual encounter sampling validates eDNA selectivity and sensitivity for the endangered wood turtle (*Glyptemys insculpta*). PLoS One.

[77] Doi H., Nakamura K. (2023). Dominant barriers and the solutions to the social application of environmental DNA. Landsc. Ecol. Eng..

[78] Yip Z.T., Quek Z.B.R., Huang D. (2025). Spatiotemporal eDNA monitoring of marine biodiversity in a hyperurbanised coastal environment. Environ. DNA.

[79] Lim L.J.W., Loh J.B.Y., Lim A.J.S., Tan B.Y.X., Ip Y.C.A., Neo M.L., Tan R., Huang D. (2020). Diversity and distribution of intertidal marine species in Singapore. Raffles Bull. Zool..

[80] Chou L.M., Huang D., Tan K.S., Toh T.C., Goh B.P.L., Tun K. (2019). Singapore World Seas: An Environmental Evaluation. Volume II: The Indian Ocean to the Pacific.

[81] Lai S., Loke L.H.L., Hilton M.J., Bouma T.J., Todd P.A. (2015). The effects of urbanisation on coastal habitats and the potential for ecological engineering: A Singapore case study. Ocean Coast Manag..

[82] McKenzie L.J., Yaakub S.M., Tan R., Seymour J., Yoshida R.L. (2016). Seagrass habitats of Singapore: Environmental drivers and key processes. Raffles Bull. Zool..

[83] Rourke M.L., Fowler A.M., Hughes J.M., Broadhurst M.K., DiBattista J.D., Fielder S., Wilkes Walburn J., Furlan E.M. (2022). Environmental DNA (eDNA) as a tool for assessing fish biomass: A review of approaches and future considerations for resource surveys. Environ. DNA.

[84] Bessey C., Neil Jarman S., Simpson T., Miller H., Stewart T., Kenneth Keesing J., Berry O. (2021). Passive eDNA collection enhances aquatic biodiversity analysis. Commun. Biol..

[85] Chen X., Kong Y., Zhang S., Zhao J., Li S., Yao M. (2022). Comparative evaluation of common materials as passive samplers of environmental DNA. Environ. Sci. Technol..

[86] Jeunen G., von Ammon U., Cross H., Ferreira S., Lamare M., Day R., Treece J., Pochon X., Zaiko A., Gemmell N.J. (2022). Moving environmental DNA (eDNA) technologies from benchtop to the field using passive sampling and PDQeX extraction. Environ. DNA.

[87] Simmons M., Tucker A., Chadderton W.L., Jerde C.L., Mahon A.R. (2016). Active and passive environmental DNA surveillance of aquatic invasive species. Can. J. Fish. Aquat. Sci..

[88] Gin K.Y.H., Lin X., Zhang S. (2000). Dynamics and size structure of phytoplankton in the coastal waters of Singapore. J. Plankton Res..

[89] Jeunen G., Lamare M.D., Knapp M., Spencer H.G., Taylor H.R., Stat M., Bunce M., Gemmell N.J. (2020). Water stratification in the marine biome restricts vertical environmental DNA (eDNA) signal dispersal. Environ. DNA.

[90] Monuki K., Barber P.H., Gold Z. (2021). eDNA captures depth partitioning in a kelp forest ecosystem. PLoS One.

[91] Reef R., Atwood T.B., Samper-Villarreal J., Adame M.F., Sampayo E.M., Lovelock C.E. (2017). Using eDNA to determine the source of organic carbon in seagrass meadows. Limnol. Oceanogr..

[92] McCartin L.J., Vohsen S.A., Ambrose S.W., Layden M., McFadden C.S., Cordes E.E., McDermott J.M., Herrera S. (2022). Temperature controls eDNA persistence across physicochemical conditions in seawater. Environ. Sci. Technol..

[93] Saito T., Doi H. (2021). A model and simulation of the influence of temperature and amplicon length on environmental DNA degradation rates: a meta-analysis approach. Front. Ecol. Evol..

[94] Tzafesta E., Shokri M. (2025). The combined negative effect of temperature, UV radiation and salinity on eDNA detection: A global meta-analysis on aquatic ecosystems. Ecol. Indic..

[95] Harrison J.B., Sunday J.M., Rogers S.M. (2019). Predicting the fate of eDNA in the environment and implications for studying biodiversity. Proc. R. Soc. B.

[96] Sirois S.H., Buckley D.H. (2019). Factors governing extracellular DNA degradation dynamics in soil. Environ. Microbiol. Rep..

[97] Pawlowski J., Bruce K., Panksep K., Aguirre F.I., Amalfitano S., Apothéloz-Perret-Gentil L., Baussant T., Bouchez A., Carugati L., Cermakova K. (2022). Environmental DNA metabarcoding for benthic monitoring: A review of sediment sampling and DNA extraction methods. Sci. Total Environ..

[98] Ørberg S.B., Krause-Jensen D., Geraldi N.R., Ortega A., Díaz-Rúa R., Duarte C.M. (2022). Fingerprinting Arctic and North Atlantic macroalgae with eDNA – application and perspectives. Environ. DNA.

[99] Mubarak M., Rifardi R., Nurhuda A., Syahputra R.F., Retnawaty S.F. (2022). Sea surface temperature (SST) and rainfall trends in the Singapore Strait from 2002 to 2019. Indones. J. Geogr..

[100] Revéret A., Rijal D.P., Heintzman P.D., Brown A.G., Stoof-Leichsenring K.R., Alsos I.G. (2023). Environmental DNA of aquatic macrophytes: The potential for reconstructing past and present vegetation and environments. Freshw. Biol..

[101] Shaltout M. (2019). Recent sea surface temperature trends and future scenarios for the Red Sea. Oceanologia.

[102] DiBattista J.D., Berumen M.L., Priest M.A., De Brauwer M., Coker D.J., Sinclair-Taylor T.H., Hay A., Bruss G., Mansour S., Bunce M. (2022). Environmental DNA reveals a multi-taxa biogeographic break across the Arabian Sea and Sea of Oman. Environ. DNA.

[103] van Maren D.S., Gerritsen H. (2012). Residual flow and tidal asymmetry in the Singapore Strait, with implications for resuspension and residual transport of sediment. J. Geophys. Res..

[104] Wee A.K.S., Salmo Iii S.G., Sivakumar K., Then A.Y.-H., Basyuni M., Fall J., Habib K.A., Isowa Y., Leopardas V., Peer N. (2023). Prospects and challenges of environmental DNA (eDNA) metabarcoding in mangrove restoration in Southeast Asia. Front. Mar. Sci..

[105] Curtis A.N., Tiemann J.S., Douglass S.A., Davis M.A., Larson E.R. (2021). High stream flows dilute environmental DNA (eDNA) concentrations and reduce detectability. Divers. Distrib..

[106] Huerlimann R., Cooper M.K., Edmunds R.C., Villacorta-Rath C., Le Port A., Robson H.L.A., Strugnell J.M., Burrows D., Jerry D.R. (2020). Enhancing tropical conservation and ecology research with aquatic environmental DNA methods: an introduction for non-environmental DNA specialists. Anim. Conserv..

[107] Jane S.F., Wilcox T.M., McKelvey K.S., Young M.K., Schwartz M.K., Lowe W.H., Letcher B.H., Whiteley A.R. (2015). Distance, flow and PCR inhibition: eDNA dynamics in two headwater streams. Mol. Ecol. Resour..

[108] Lau K.-M., Wu H.-T. (2007). Detecting trends in tropical rainfall characteristics, 1979–2003. Int. J. Climatol..

[109] Akatsuka M., Takayama Y., Muchebve E., Ito K., Kuwae T., Watanabe K., Minamoto T. (2020). Proceedings of the 22nd IAHR-APD Congress 2020.

[110] Muchebve E., Takayama Y., Akatsuka M., Ito K., Minamoto T. (2020). Feasibility study for seagrass beds monitoring using environmental DNA. Journal of Japan Society of Civil Engineers, Ser. B2 (Coastal Engineering).

[111] IPCC (2021).

[112] Lacombe G., Hoanh C.T., Smakhtin V. (2012). Multi-year variability or unidirectional trends? Mapping long-term precipitation and temperature changes in continental Southeast Asia using PRECIS regional climate model. Clim. Change.

[113] Loo Y.Y., Billa L., Singh A. (2015). Effect of climate change on seasonal monsoon in Asia and its impact on the variability of monsoon rainfall in Southeast Asia. Geosci. Front..

[114] Su H., Wu L., Zhai C., Jiang J.H., Neelin J.D., Yung Y.L. (2020). Observed tightening of tropical ascent in recent decades and linkage to regional precipitation changes. Geophys. Res. Lett..

[115] Lamb P.D., Fonseca V.G., Maxwell D.L., Nnanatu C.C. (2022). Systematic review and meta-analysis: Water type and temperature affect environmental DNA decay. Mol. Ecol. Resour..

[116] Lovelock C.E., Reef R. (2020). Variable impacts of climate change on blue carbon. One Earth.

[117] Duarte C.M., Gattuso J., Hancke K., Gundersen H., Filbee-Dexter K., Pedersen M.F., Middelburg J.J., Burrows M.T., Krumhansl K.A., Wernberg T. (2022). Global estimates of the extent and production of macroalgal forests. Global Ecol. Biogeogr..

[118] Skelton J., Cauvin A., Hunter M.E. (2023). Environmental DNA metabarcoding read numbers and their variability predict species abundance, but weakly in non-dominant species. Environ. DNA.

[119] Ledger K.J., Hicks M.B.R., Hurst T.P., Larson W., Baetscher D.S. (2024). Validation of environmental DNA for estimating proportional and absolute biomass. Environ. DNA.

[120] Holman L.E., Chng Y., Rius M. (2022). How does eDNA decay affect metabarcoding experiments?. Environ. DNA.

[121] Ip Y.C.A., Tay Y.C., Gan S.X., Ang H.P., Tun K., Chou L.M., Huang D., Meier R. (2019). From marine park to future genomic observatory? Enhancing marine biodiversity assessments using a biocode approach. Biodivers. Data J..

[122] Wang Y., Pedersen M.W., Alsos I.G., De Sanctis B., Racimo F., Prohaska A., Coissac E., Owens H.L., Merkel M.K.F., Fernandez-Guerra A. (2021). Late Quaternary dynamics of Arctic biota from ancient environmental genomics. Nature.

[123] Weigand H., Beermann A.J., Čiampor F., Costa F.O., Csabai Z., Duarte S., Geiger M.F., Grabowski M., Rimet F., Rulik B. (2019). DNA barcode reference libraries for the monitoring of aquatic biota in Europe: Gap-analysis and recommendations for future work. Sci. Total Environ..

[124] Kwan V., Yip Z.T., Fong J., Huang D. (2021). Diversity and phylogeny of the brown alga *Lobophora* (Dictyotales, Phaeophyceae) in Singapore. Phytotaxa.

[125] Ng Y.F., Huang D. (2024). Species diversity and phylogeny of the green macroalga *Ulva* (Ulvophyceae, Chlorophyta) in Singapore. Phytotaxa.

[126] Guardiola M., Uriz M.J., Taberlet P., Coissac E., Wangensteen O.S., Turon X. (2015). Deep-sea, deep-sequencing: metabarcoding extracellular DNA from sediments of marine canyons. PLoS One.

[127] Zhang J., Kobert K., Flouri T., Stamatakis A. (2014). PEAR: a fast and accurate Illumina Paired-End reAd mergeR. Bioinformatics.

[128] Boyer F., Mercier C., Bonin A., Le Bras Y., Taberlet P., Coissac E. (2016). OBITOOLS: a UNIX-inspired software package for DNA metabarcoding. Mol. Ecol. Resour..

[129] Jeunen G.J., Dowle E., Edgecombe J., Von Ammon U., Gemmell N.J., Cross H. (2023). CRABS—A software program to generate curated reference databases for metabarcoding sequencing data. Mol. Ecol. Resour..

[130] Wood D.E., Lu J., Langmead B. (2019). Improved metagenomic analysis with Kraken 2. Genome Biol..

[131] Camacho C., Coulouris G., Avagyan V., Ma N., Papadopoulos J., Bealer K., Madden T.L. (2009). BLAST+: architecture and applications. BMC Bioinform.

[132] Katoh K., Standley D.M. (2013). MAFFT multiple sequence alignment software version 7: improvements in performance and usability. Mol. Biol. Evol..

[133] Meier R., Shiyang K., Vaidya G., Ng P.K.L. (2006). DNA barcoding and taxonomy in Diptera: a tale of high intraspecific variability and low identification success. Syst. Biol..

[134] Meier R., Wong W., Srivathsan A., Foo M. (2016). $1 DNA barcodes for reconstructing complex phenomes and finding rare species in specimen-rich samples. Cladistics.

[135] R Core Team (2013). R: A Language and Environment for Statistical Computing;. https://cran.r-project.org.

[136] Oksanen J., Blanchet F.G., Kindt R., Legendre P., Minchin P.R., O’Hara R.B., Simpson G.L., Solymos P., Stevens M.H.H., Wagner H. (2013). Vegan: Community Ecology Package. R Package Version 2.0-8.

[137] Martinez Arbizu P. (2020). PairwiseAdonis: Pairwise multilevel comparison using adonis. R Package Version 0.4.

[138] Lim T.K., Chou L.M. (2020). Pulau Semakau Landfill – A haven for coastal and marine biodiversity. ENVISION Magazine.

[139] Teo S., Yeo R.K.H., Chong K.Y., Chung Y.F., Neo L., Tan H.T.W. (2011). The flora of Pulau Semakau: A Project Semakau checklist. Nature in Singapore.

[140] Galanis A., Vardakas P., Reczko M., Harokopos V., Hatzis P., Skoulakis E.M.C., Pavlopoulos G.A., Patalano S. (2022). Bee foraging preferences, microbiota and pathogens revealed by direct shotgun metagenomics of honey. Mol. Ecol. Resour..

[141] Wright R.J., Comeau A.M., Langille M.G.I. (2023). From defaults to databases: parameter and database choice dramatically impact the performance of metagenomic taxonomic classification tools. Microb. Genom..

[142] Quast C., Pruesse E., Yilmaz P., Gerken J., Schweer T., Yarza P., Peplies J., Glöckner F.O. (2013). The SILVA ribosomal RNA gene database project: improved data processing and web-based tools. Nucleic Acids Res..

[143] Yilmaz P., Parfrey L.W., Yarza P., Gerken J., Pruesse E., Quast C., Schweer T., Peplies J., Ludwig W., Glöckner F.O. (2014). The SILVA and “All-species Living Tree Project (LTP)” taxonomic frameworks. Nucleic Acids Res..

[144] Lee A.C., Liao L.M., Tan K.S. (2009). New records of marine algae on artificial structures and intertidal flats in coastal waters of Singapore.. Raffles Bull. Zool..

[145] Lee A.C., Baula I.U., Miranda L.N., Sin T.M., Sin T.M., Wang L.K. (2015). Tropical Marine Science Institute.

[146] Lee A.C., Sin T.M. (2009). Intertidal assemblages on coastal defence structures in Singapore II. Contrasts between islands and the mainland. Raffles Bull. Zool..

[147] Noiraksar T., Lewmanomont K., Tan K.S., Ong J.J.L. (2012). Contributions to Marine Science.

[148] Phang V.X.H., Chou L.M., Friess D.A. (2015). Ecosystem carbon stocks across a tropical intertidal habitat mosaic of mangrove forest, seagrass meadow, mudflat and sandbar. Earth Surf. Process. Landf..

[149] Harris D., Horwáth W.R., Van Kessel C. (2001). Acid fumigation of soils to remove carbonates prior to total organic carbon or CARBON-13 isotopic analysis. Soil Sci. Soc. Am. J..

[150] Laporte M., Reny-Nolin E., Chouinard V., Hernandez C., Normandeau E., Bougas B., Côté C., Behmel S., Bernatchez L. (2021). Proper environmental DNA metabarcoding data transformation reveals temporal stability of fish communities in a dendritic river system. Environ. DNA.

