# Supplementary material for: Characterizing the diversity and distribution of tropical coastal blue carbon using environmental DNA
Source: iScience. 2025 Oct 24;28(11):113837. doi: 10.1016/j.isci.2025.113837 (PMC12682276; doi:10.1016/j.isci.2025.113837)
Supplement: Document S1. Figures S1–S6 [file mmc1.pdf]

iScience, Volume *28*

Supplemental information

Characterizing the diversity and distribution of tropical coastal blue carbon using environmental DNA

Wei Jie Dennis Tan, Jia Jin Marc Chang, Valerie Kwan, and Danwei Huang

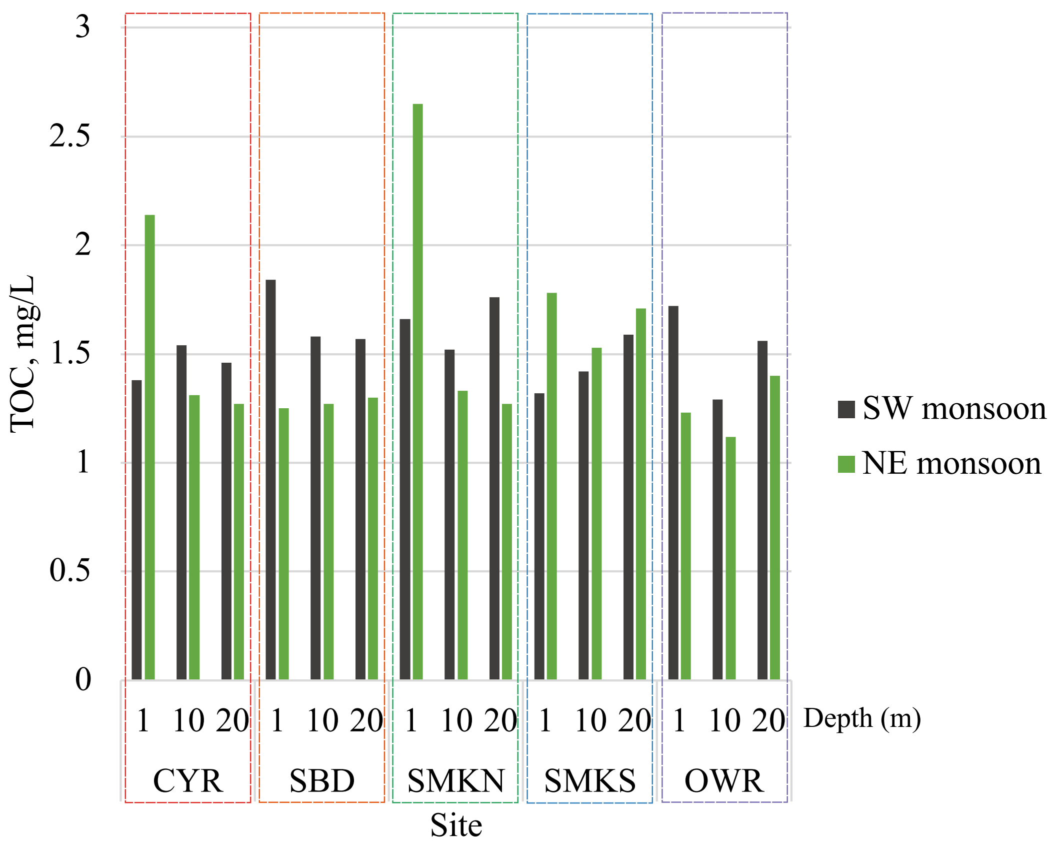

Figure S1. Water TOC levels measured at each study site during the southwest (SW) and northeast (NE) monsoons.

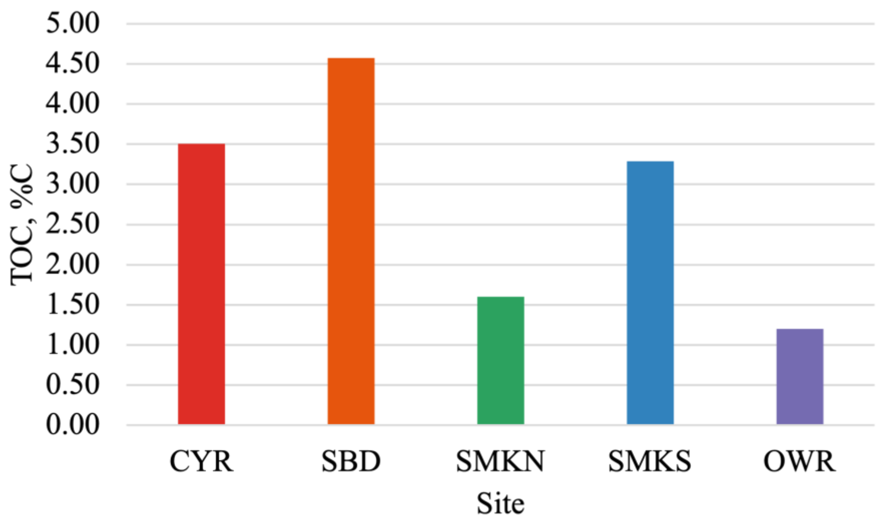

Figure S2. Sediment TOC levels measured at each study site during the southwest monsoon.

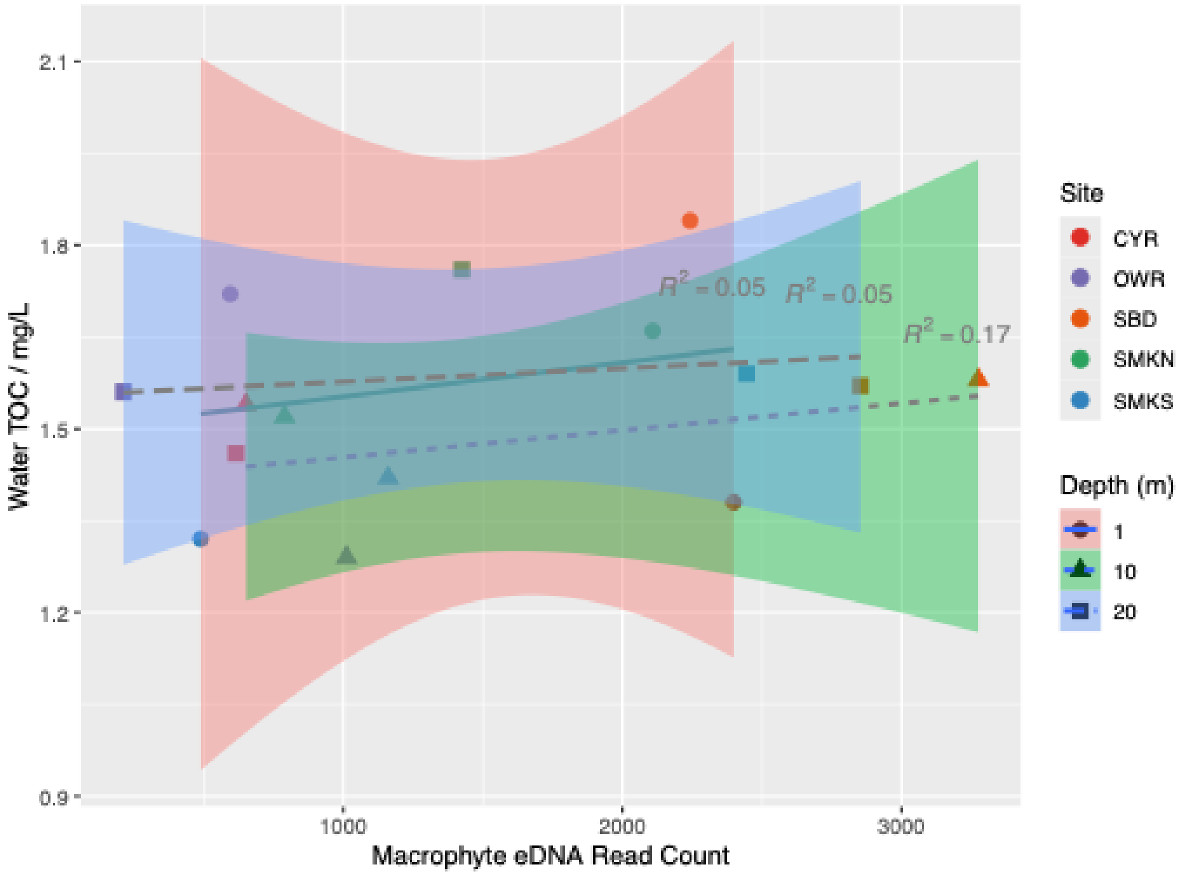

Figure S3. Total organic carbon (TOC) levels in water samples collected during the southwest monsoon at each site and depth against the abundance of macrophyte eDNA reads. Lines represent linear regression at each sampling depth.

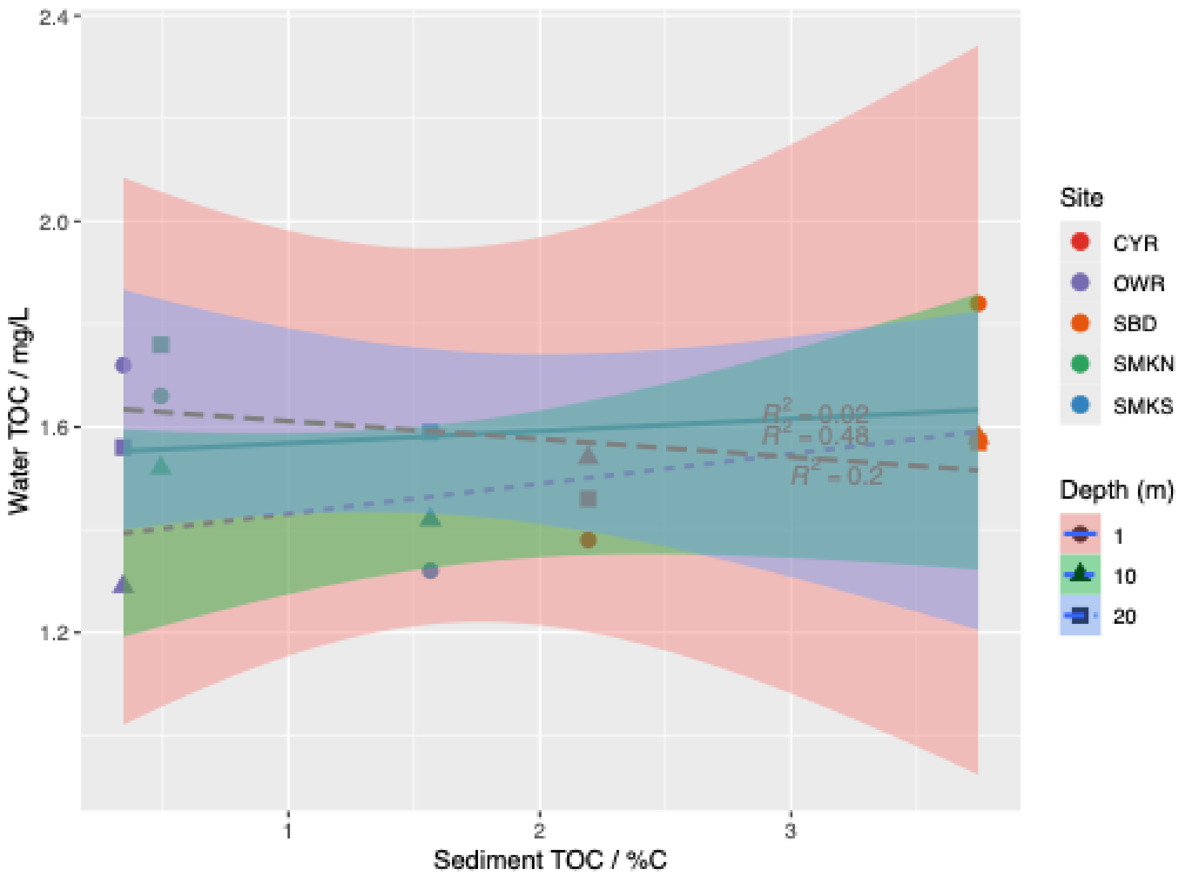

Figure S4. Total organic carbon (TOC) levels in water samples collected during the southwest monsoon at each site and depth against the TOC levels in sediment samples. Lines represent linear regression at each water depth.

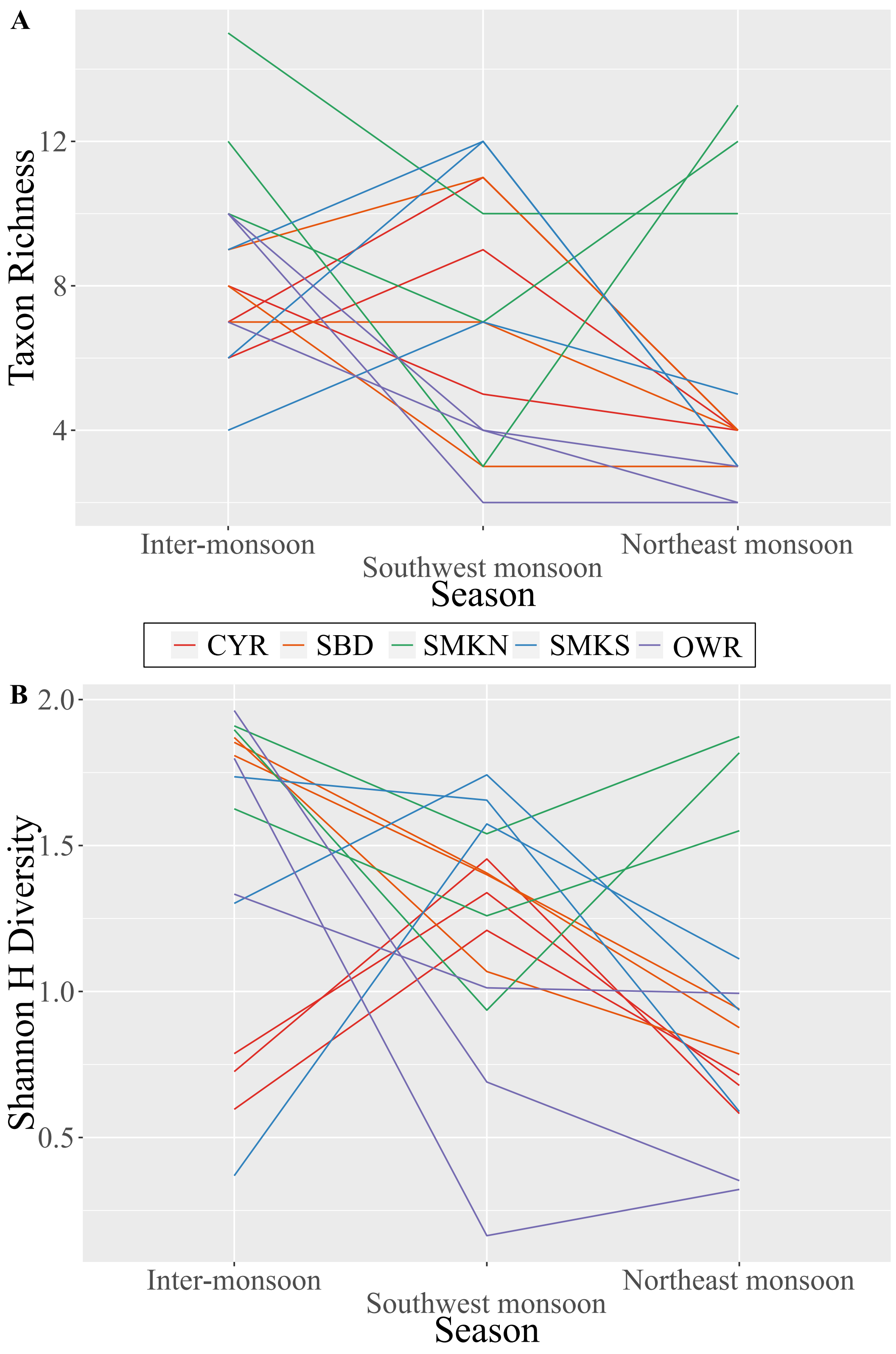

Figure S5. Variations in coastal macrophyte richness (A) and diversity (B) at each study site among seasons. Each site is represented by three lines, one for each sampling depth.

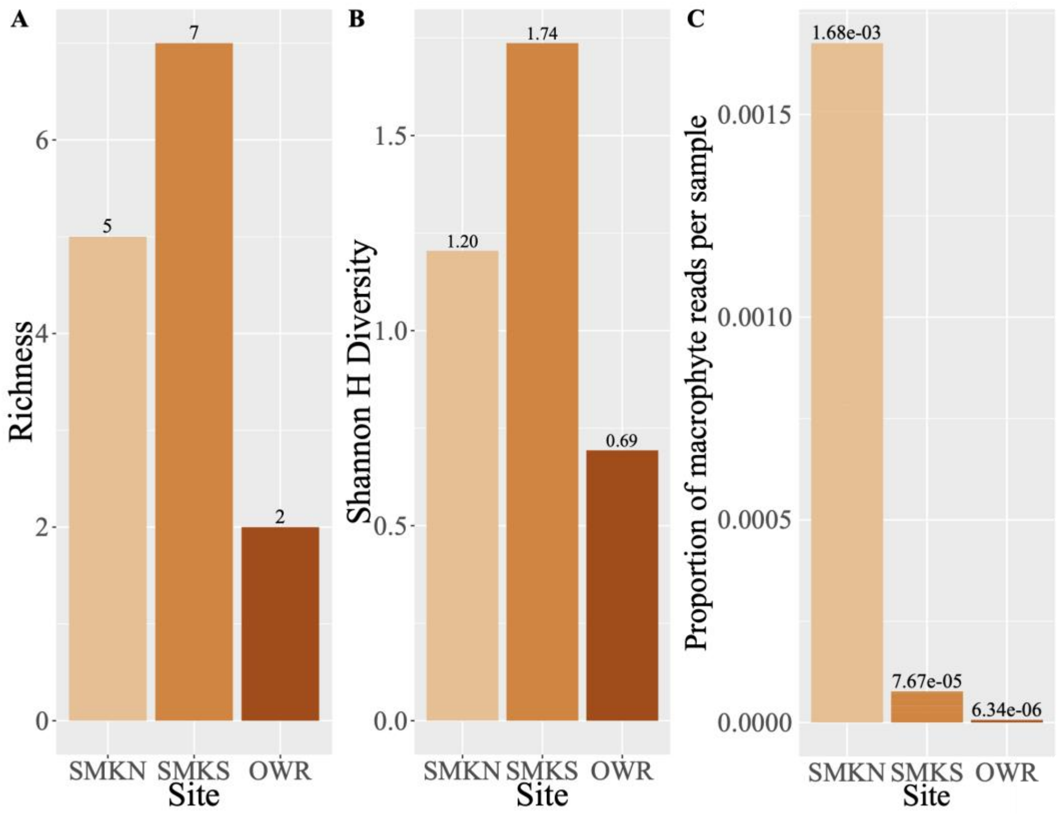

Figure S6. Coastal macrophyte diversity in sediment collected during the inter-monsoon at three study sites by richness (A), diversity (B), and proportional abundance of macrophyte reads (C).
